# Suppression treatment differentially influences the microbial community and the occurrence of broad host range plasmids in the rhizosphere of the model cover crop *Avena sativa* L.

**DOI:** 10.1371/journal.pone.0223600

**Published:** 2019-10-09

**Authors:** Marco Allegrini, Elena del V. Gomez, Kornelia Smalla, María Celina Zabaloy

**Affiliations:** 1 Laboratorio de Biodiversidad Vegetal y Microbiana, Campo Experimental J. Villarino, Instituto de Investigaciones en Ciencias Agrarias de Rosario (IICAR CONICET-UNR), Universidad Nacional de Rosario, Zavalla, Argentina; 2 Institute for Epidemiology and Pathogen Diagnostics, Federal Research Centre for Cultivated Plants (JKI), Julius Kühn-Institut, Braunschweig, Germany; 3 Centro de Recursos Naturales Renovables de la Zona Semiárida (CERZOS), Universidad Nacional del Sur (UNS)-CONICET, Bahía Blanca, Argentina; 4 Departamento de Agronomía, Universidad Nacional del Sur, Bahía Blanca, Argentina; University of Bath, UNITED KINGDOM

## Abstract

Cover crop suppression with glyphosate-based herbicides (GBHs) represents a common agricultural practice. The objective of this study was to compare rhizospheric microbial communities of *A*. *sativa* plants treated with a GBH relative to the mechanical suppression (mowing) in order to assess their differences and the potential implications for soil processes. Samples were obtained at 4, 10, 17 and 26 days post-suppression. Soil catabolic profiling and DNA-based methods were applied. At 26 days, higher respiration responses and functional diversity indices (Shannon index and catabolic evenness) were observed under glyphosate suppression and a neat separation of catabolic profiles was detected in multivariate analysis. Sarcosine and Tween 20 showed the highest contribution to this separation. Metabarcoding revealed a non-significant effect of suppression method on either alpha-diversity metrics or beta-diversity. Conversely, differences were detected in the relative abundance of specific bacterial taxa. *Mesorhizobium* sequences were detected in higher relative abundance in glyphosate-treated plants at the end of the experiment while the opposite trend was observed for *Gaiella*. Quantitative PCR of *amoA* gene from ammonia-oxidizing archaea showed a lower abundance under GBH suppression again at 26 days, while ammonia-oxidizing bacteria remained lower at all sampling times. Broad host range plasmids IncP-1β and IncP-1ε were exclusively detected in the rhizosphere of glyphosate-treated plants at 10 days and at 26 days, respectively. Overall, our study demonstrates differential effects of suppression methods on the abundance of specific bacterial taxa, on the physiology and mobile genetic elements of microbial communities while no differences were detected in taxonomic diversity.

## Introduction

Cover crops (CC) have gained popularity in agricultural practices as a sustainable alternative to fallow between grain crops, to incorporate carbon (C) rich residues in soils and to promote soils coverage, reducing risks of erosion, nitrogen (N) losses, and weeds proliferation [1,2,3]. Among them, rye (*Secale cereale* L.), oat (*Avena sativa* L.), barley (*Hordeum vulgare* L.), ryegrass (*Lolium multiflorum* L.) or triticale (*Triticosecale* Wittm. ex A. Camus) are the main species. The CC is necessarily killed to allow sowing the cash crop and the broad spectrum and non-selective herbicide glyphosate (*N*-[phosphonomethyl]glycine) is mostly used to achieve this purpose. Cover crop suppression with glyphosate-based herbicides (GBHs) represents a common agricultural practice.

Considering the growing trend of CC in sustainable agriculture, thoroughly assessing the effects of suppression managements on microbial communities deserves investigation. With regards to chemical dessication, several studies have demonstrated that glyphosate can be translocated to roots and subsequently released to the surrounding soil [4–8]. Glyphosate may also modify the quality and quantity of rhizodeposits stimulating the exudation of carbohydrates and amino acids [6,9] and promoting the turnover of dead root tissue [10], which, in turn, induce shifts in rhizospheric microbial communities [10]. The use of mechanical methods (mowing or rolling) is less extended [1,3]. As for them, no effects have been observed on bacterial communities (16S rRNA gene copy numbers) after mowing of barley [10] and on community structure of fungal/bacterial communities (assessed by denaturing gradient electrophoresis, DGGE) after defoliation of ryegrass [11]. The microbial activity and the proportion of fast and slow-growing bacteria were also not affected by mowing treatment in barley [10].

Different rhizospheric microbial communities assembled in the decaying root material, following glyphosate desiccation as opposed to herbicide-free mechanical suppression, could have a differential influence on soil functions, a matter which deserves further research. This consideration is particularly relevant for microbial groups involved in ecologically relevant processes after CC suppression, like the turnover of rhizodeposits and decaying organic material (*Actinobacteria*) and N-cycling of mineralized N (ammonia-oxidizing microorganisms). Previous studies in other crops have investigated the differences in rhizospheric microbial communities under glyphosate suppression relative to mechanical treatments [10,12]. Significantly higher values of functional diversity parameters (Shannon index and richness) were observed for microbial communities of glyphosate-treated triticale compared to cut plants 15 days after treatment, while no differences were observed in Shannon diversity index from 16S rDNA PCR-DGGE fingerprints [12]. Similarly, a higher number of culturable bacteria and most probable number of protists were observed in the rhizosphere of glyphosate-treated barley plants in comparison to cut plants [10].

The rhizosphere is considered a survival hotspot for mobile genetic elements, hosting a microbiome prone to horizontal gene transfer. The high densities of physiologically active microbial cells along with the fluxes of nutrients in the rhizodeposits are favorable for increased rates of DNA exchange [13,14], e.g., through the conjugative transfer of broad host range plasmids of IncP-1 group commonly associated to antibiotic resistance and catabolic genes [15,16]. As stated above, increased rhizodeposition in glyphosate-treated plants stimulates microbial activity in the rhizosphere, which in turn may facilitate IncP-1 plasmids mobilization, an uninvestigated matter.

The objective of our study was to compare rhizospheric microbial communities of *A*. *sativa* plants suppressed with a GBH relative to the mechanical suppression (mowing), through a multiple-methodology approach, in order to gain a clearer understanding of which microbial groups could differ and the implications for soil processes. To achieve this goal, we employed high-throughput sequencing of bacterial 16S rRNA gene (metabarcoding), profiled the respiratory response to different C sources, searched for broad host range plasmids and quantitated the abundances of phylogenetic and functional-sensitive microbial groups linked to ecologically relevant functions such as ammonia-oxidizing microorganisms and *Actinobacteria*. In the light of current knowledge, we hypothesize that differential effects of suppression methods on rhizodeposition would be detectable at the microbial community level in the rhizosphere as rhizodeposits are key drivers of the structure and function of microbial communities [14]. Specifically, we expect that glyphosate-treated plants exudation will stimulate the respiratory response of associated rhizospheric microbial communities while also promoting catabolic diversity. We also expect a higher abundance of IncP-1 plasmids as a result of this higher microbial activity and a lower abundance of ammonia-oxidizing microorganisms and *Actinobacteria* due to their sensitivity as soil microbiological indicators [17]. Regarding diversity, we expect no overall differences in taxonomic diversity [12] although species composition may change.

## Material and methods

### Experimental design

A short-term greenhouse experiment was conducted under a factorial completely randomized design. The experimental factors were two suppression methods (“M”) (mowing and glyphosate) × four sampling time-points (“S”) (4, 10, 17, 26 days after suppression), resulting in a total of 8 treatment-combinations (Table 1).

**Table 1:** Design of treatments in the experiment. A factorial design was considered with two factors under study: suppression method (M) and sampling time (S). The labels of the eight treatment-combinations, with four replicates each (n = 4), are indicated.

Sampling time (S)	Suppression method (M)	
	Mowing (C)	Glyphosate (G)
4 days	C.4D	G.4D
10 days	C.10D	G.10D
17 days	C.17D	G.17D
26 days	C.26D	G.26D

In September 2015, 15 cores of bulk soil (0–15 cm) were sampled from a 625 m^2^ area in a plot with more than 20 years of history of GBHs (33°02'23''S, 60°53'05''W) and mixed in a composite sample after sieving (< 6 mm). The soil is a Vertic Argiudoll (Ap horizon: clay 406 g kg^−1^, silt 491 g kg^−1^, sand 103 g kg^−1^, organic matter 44.1 g kg^−1^, pH(H_2_O): 5.5). The composite sample was used immediately for preparation of experimental 3 L pots with 1.8 kg of soil and perlite at 80:20 v/v ratio (soil:perlite). In these pots, seeds of *Avena sativa* L. var. Cristal INTA were sown. After germination plants were grown in the greenhouse for 67 days (vegetative stage) under the following conditions: temperature 16/28°C (average minimum/maximum day temperature), 13/11 light/dark hours on average according to the growing period (September to November) (natural light, without additional light) and watered by capillarity using individual trays for each pot containing tap water. At 67 days after sowing, the following suppression methods were applied: 1) Mowing of the aerial part of the plant at 1 cm from the soil surface using a sharp scissor 2) Chemical desiccation of plants with a commercial formulation of glyphosate sprayed at the recommended field rate (4 L ha^-1^, Eskoba Full II, Red Surcos, 662 g L^-1^, monopotassium salt) using a hand-held sprayer. The area of the pot was considered in order to calculate the exact volume of commercial formulation required per pot. The herbicide was prepared at the moment of application, by dissolving the calculated volume of the product in 4 mL of distilled water per pot. Other studies have used a similar spraying volume [7]. We selected this volume as a trade-off between an appropriate small spray volume to cover the foliage without leaks by runoff from leaf surface and an appropriate dilution volume of the surfactant per unit of area. A higher dilution of the surfactant could reduce the efficiency of the herbicide [18].

Sampling of rhizospheric soil was conducted destructively at 4, 10, 17 and 26 days after plant suppression. At each sampling time, soil samples were collected and analyzed from four replicates (four pots) per suppression method. In order to consider plant variability in each pot, five plants were removed and rhizospheric soil was sampled. Loosely adhering soil (removed by gentle shaking of the root system) was considered as bulk soil. The tightly adhering soil (approx. 0–5 mm), removed by brushing the root system, was considered as rhizospheric soil [19]. The five samples of rhizospheric soil of the pot were mixed in equal quantities (composite sample of the pot), a procedure previously reported in the rhizosphere of glyphosate-treated plants [20]. The soil was sieved (< 2 mm) and the composite samples were divided in two fractions: one was stored at -80°C for molecular analysis and the other was used immediately for physiological analysis.

### Collection of root exudates

After separation of rhizospheric soil, the root systems from glyphosate-treated or cut plants were used immediately for collection of root exudates. For each treatment (e.g. C.4D), the roots from the five plants in each of the four replicate pots (n = 4) were pooled (20 plants per treatment) and used for collection of exudates, according to the protocol described by Egle et al. [21]. Firstly, the root system was completely and carefully washed with tap water to remove all mineral particles attached. Then, the roots were submerged completely in an Erlenmeyer flask containing 200 mL of collection solution (CaCl_2_ 0.05 mM, pH 5.5) for 1 h in order to detach possibly damaged cells during the removal of soil and the washing step. The liquid was discarded and the roots were submerged again in 200 mL of the collection solution for 4 h (always in the afternoon) in the same conditions in which plants were grown. In order to assess the time-specific response of microbial communities, root exudates were collected at 4, 10, 17 and 26 days and tested separately with the corresponding soil samples collected at the same sampling time (see next section). The exudates were sterilized using Minisart filters (0.22 μm).

### Physiological analysis of microbial communities

#### Catabolic profiles

Catabolic profiles of microbial communities were obtained by measuring oxygen consumption of a soil suspension with the fluorimetric 96 wells-microplate system BD Oxygen BioSensor System (BDOBS) [22], after the addition of 9 substrates (described below) or the root exudates (obtained as mentioned previously). Substrates (Sigma) were selected to cover a range of chemical compounds: amino acids (L-phenylalanine, L-asparagine, N-methyl glycine or sarcosine), carbohydrates (D-xylose, D-cellobiose), organic acids (sodium pyruvate and sodium fumarate), *p*-coumaric acid and the surfactant polyoxyethylene (20) sorbitan monolaurate (Tween 20, Promega). In addition, the selection was based on significant differences reported for L-phenylalanine, L-asparagine, D-xylose and D-cellobiose and surfactants from Tween family in the rhizosphere of triticale between glyphosate-treated and clipped plants [12]. Tween 20 is an ethoxylated surfactant. Ethoxylated adjuvants are the most common additives found in different agrochemical formulations [23,24], although no information about additives are provided in the commercial formulation used in this study. Coumaric acid is an ecologically relevant phenylpropanoid related with lignin degradation. Significant differences in the respiratory response to this substrate have been reported [25,26].

The final concentration of substrates in the plate was 50 mg L^-1^, except for Tween 20 (3 ppm). Basal respiration (BR) was measured by loading the same volume (100 μL) of filter sterilized distilled water instead of substrate addition. Similarly, the respiratory response in the presence of root exudates from glyphosate-treated or cut plants was assessed. Soil suspensions and substrate solutions were prepared in sterile distilled water and 200 μL of soil suspension (1:7.5 soil:water ratio) was loaded in each well. At each sampling time, a full 96-well BDOBS plate was used: 12 columns (9 substrates + 2 root exudates + distilled water) × 8 rows (4 biological replicates per suppression method).

The kinetic measures of fluorescence (relative fluorescence units, RFU) were recorded from the bottom of the microplate (bottom-reading mode) every 15 min for 24 h in a microplate fluorometer FLUOstar Optima (BMG Labtech, Offenburg, Germany) at constant temperature of 30°C, using a 470 nm filter for excitation and a 610 nm emission filter. Readings at each time point (RFU) were divided by the response at 1 h to express data as normalized relative fluorescence units (NRFU), considering a delayed time point for normalization in order to allow for temperature equilibration given the temperature sensitivity of the ruthenium dye. NRFU was plotted vs. time (hours) to obtain respiratory curves. The integrated area under the respiratory curve (AUC) was calculated between 1 and 6 h with the software SigmaPlot 10.0 (Systat Software, Inc., San Jose, CA, USA) [27]. This initial period of time was selected to include only the respiratory response of non-growing populations according to substrate induced respiration (SIR) definition [28]. The SIR response and the BR were used for calculation of a carbon availability index (CAI; CAI = BR / SIR) according to Cheng et al. [29].

#### Functional diversity

Catabolic diversity was assessed through calculation of Shannon Index (*H*’), similarly to Mijangos et al. [12]:

$$H’=-\sum p_{i}log_{2}p_{i}$$

*i* = 1…*s*; *s* = number of substrates; *p*_*i*_ = respiratory response (AUC) with *i*-esime substrate (*r*_*i*_) relative to the response to all (∑*r*_*i*_) substrates (*p*_*i*_ = *r*_*i*_/∑ *r*_*i*_).

In addition to Shannon Index, catabolic evenness (*E*) was also calculated. Catabolic evenness is defined as a component of microbial functional diversity which measures the uniformity of substrate use and was calculated according to the formula reported by Degens et al. [30]:

$$E=1/\sum p_{i}^{2},$$

with *p*_*i*_ summed for all substrates.

### DNA-based analysis of microbial communities

#### DNA extraction and quantification

The commercial kit PowerSoil^™^ DNA Isolation kit (MoBio, Inc., Carlsbad, CA) was used for DNA extraction and purification from 250 mg of rhizospheric soil according to manufacturer instructions. The kit includes a bead beating tube for rapid and thorough homogenization. Cell lysis occurs by mechanical and chemical method. DNA was quantified using QuantiFluor dsDNA kit in a Quantus fluorometer (Promega).

#### Metabarcoding analysis: Library preparation and data processing

The following procedure was conducted with DNA samples from both suppression methods collected at the first and last sampling time. Three replicates were analyzed in each case.

Pyrosequencing of amplicons from 16S rRNA gene was performed by Molecular Research MR DNA (Shallowater, Texas, USA), using 454 FLX Titanium technologies (Roche) according to bTEFAP^™^ procedure [31] with 400 bp pyrotags and a sequencing depth of 3000 nominal reads per sample. For library construction, amplicons were obtained using the universal primer pair 515F and 806R directed toward V4 region of 16S rRNA gene (S1 Table). These primers yield good coverage and accurate phylogenetic information with no significant bias against any bacterial phyla, including the underestimated phylum *Verrucomicrobia* [32]. A single-step 30 cycle PCR was conducted using HotStarTaq Plus Master Mix Kit (Qiagen, Valencia, CA) under the following PCR program: 94°C for 3 min, followed by 28 cycles of 94°C for 30 seconds; 53°C for 40 seconds and 72°C for 1 min; the final elongation step was set at 72°C for 5 min. All amplicon products from different samples were mixed in equal concentrations previous to Agencourt Ampure beads purification step (Agencourt Bioscience Corporation, MA, USA). Roche 454 FLX titanium instruments and reagents were used to sequence the prepared library following manufacturer’s guidelines.

Q25 sequence data (quality score 25) was processed using a proprietary analysis pipeline from MR DNA (*www*.*mrdnalab*.*com*, MR DNA, Shallowater, TX). During data processing, sequences were depleted of barcodes and primers followed by removal of short sequences (< 150 bp), sequences with ambiguous base calls and sequences with homopolymer runs exceeding 6 bp. Sequences were then denoised, chimeras removed (UCHIME) and operational taxonomic units (OTUs) were defined by clustering at 3% divergence (97% similarity) after removal of singleton sequences [31,33,34]. Taxonomic classification was conducted in R Statistical Software v.3.5.0 [35] through *assignTaxonomy* function in *dada2* package version 1.8 [36] which provides a native implementation of the naive Bayesian classifier method [37], against a taxonomic training database derived from Ribosomal Database Project’s (RDP) (*https*:*//rdp*.*cme*.*msu*.*edu*). The *dada2*-formatted training fasta files were derived from the RDP Training Set 16 and the 11.5 release of the RDP database (https://benjjneb.github.io/dada2/training.html). The minimum bootstrap confidence for assigning a taxonomic level was 50. Rarefaction was conducted according to the sample containing the lowest number of bacterial reads (C2.26D; 1,966 reads). Rarefied data of Bacteria was used for calculation of alpha-diversity indices, allowing samples to be statistically compared through two-way ANOVA (see Statistical analysis section).

Alpha-diversity was studied through Shannon diversity index (*H*’) and the reciprocal of Simpson index (1/D):

$$H’=-\sum p_{i}ln\;p_{i}$$

*p*_*i*_ relative abundance of each OTU

$$D=\sum \left[n_{i}\left(n_{i}-1\right)\right]/\left[N\left(N-1\right)\right]$$

*n*_*i*_ = abundance of *i*-esime OTU and *N* = sample size.

Similarly, richness and evenness were assessed. In the first case, the observed number of OTUs (S’) and Chao-1 index were estimated. In the second case, two metrics were considered: Pielou’s evenness index (J’), defined as the ratio between *H*’ and *H*’_max_ (*H*’_*max*_ = *ln S*’) and Hill-ratio (*R*_1:2_) defined as the ratio between Hill numbers [38]:

$$R_{1:2}=Hill1/Hill2$$

$$Hill1=e^{H}{}^{’}$$

Hill2=1/D

Relative abundance values were compared between suppression methods at the different taxonomic levels using the statistical software STAMP (*Statistical Analysis of Metagenomic Profiles*; [39]), as described in ‘Statistical analysis’ section.

#### Quantitative PCR of different microbial groups

The effects of treatments on the estimated abundance of ammonia-oxidizing bacteria (AOB) and ammonia-oxidizing archaea (AOA) were assessed by qPCR of the corresponding *amoA* gene, encoding alpha subunit of ammonia monooxygenase enzyme (EC 1.14.99.39, AMO). Primers used for AOA [40,41] and AOB [42] are indicated in S1 Table. For Bacteria, *Actinobacteria* and Archaea 16S rRNA gene-directed primers were used [43,44,45]. As in other studies [46,47], the abundance values of these genes were used as surrogates of population sizes, although no attempt was made to convert copies into cell numbers to avoid introducing errors (e.g. errors related with an unknown number of operons per cell in mixed bacterial communities). PCR master mixes, reaction set-up and amplification programs for *amoA* gene of AOB (*amoA*_AOB_) and 16S rRNA gene of Bacteria were as described previously by Allegrini et al. [26]. Similarly, for *amoA* gene of AOA (*amoA*_AOA_) quantification was conducted according to the protocol described by Zabaloy et al. [47]. The amplification details for *Actinobacteria* and Archaea are described in S1 Text. All amplifications were conducted in ABI 7500 Real Time System (Applied Biosystems, Foster City, CA).

#### Quantitative PCR of IncP-1 plasmids

Quantification of broad host range plasmids of the IncP-1 group was attempted by qPCR according to the protocol described by Jechalke et al. [48] using two forward (F and Fz) and three reverse primers (R, Rge, Rd) targeting *korB* gene and designed to cover all variants from six IncP-1 subgroups (S1 Table). Two TaqMan probes (P and Pgz) were used [48]. All reactions were run in a Real-time PCR system (CFX Connect; Bio-Rad, Munich, Germany). Bio-Rad CFX Manager software was used for visualization of amplification curves and standard curve. A fragment of *korB* gene (amplified from pKJK5 plasmid) inserted in pGEM^®^-T cloning vector was used as standard (8.44×10^9^ copies of the plasmid μL^-1^ of standard). Decimal dilutions of the standard (10^−1^ to 10^−8^) were prepared to construct the standard curve.

#### Detection of IncP-1 plasmids from different subgroups

PCR-Southern blot hybridization was conducted to study the occurrence of IncP-1 plasmids belonging to β and ε subgroups. Firstly, *trfA* gene of IncP-1 plasmids from these subgroups was amplified with the primers developed by Bahl et al. [49] (S1 Table) following the protocol described in S1 Text. Plasmid pR751 (IncP-1β) and pKJK5 (IncP-1ε) were used as positive control in each case. Ten microliters of PCR products (281 bp) were run in 1% agarose using DIG VII (Roche) as marker. Amplicons from both suppression methods were loaded in parallel in the same gel in order to expose them to identical Southern blot conditions. After agarose gel electrophoresis (70V, 1 h), DNA was transferred by capillarity to a nylon membrane Hybond-N+ (Amersham Biosciences) using 20× SSC transfer buffer (sodium citrate 0.3 M, sodium chloride 3 M, pH 7.0) for 16 h. The PCR fragments were fixed to the membrane 2 h at 80 °C and hybridized with appropriate digoxigenin-labelled probes (Roche Diagnostic, Mannheim, Germany) according to the protocol described in S2 Text. Hybridization with the corresponding probes for IncP-1 β and ε was performed on separated membranes. Solutions and protocols for the detection of amplicons on hybridized membranes are described in S2 Text. The Digitalization of X-ray films after autoradiography was conducted on an Epson Perfection V700 Photo scanner at 300 dpi.

#### Statistical analysis

Statistical analysis of functional diversity parameters (*H’* and *E*), alpha-diversity metrics (S’, J’, *H’*, 1/D, Chao-1, *R*_1:2_) and qPCR data (log_10_ of copy number μg^-1^ DNA) was conducted in R Statistical Software v.3.5.0 [35] through two way ANOVA (α = 0.05) and Tukey’s test (α = 0.05) for *post hoc* multiple comparison of means. The significance of the F-value for M×S interaction term was considered with *P* < 0.2 [50]. In those cases, a two sample t-test (α = 0.05) with correction for unequal variances was used for comparison of suppression methods at a specific sampling time. The same statistical test (two sample t-test) was used in physiological profiles for comparison of means of SIR measures. In all cases, normality was verified through modified Shapiro-Wilks test and the homoscedasticity in two-way ANOVA through Levene test (α = 0.01).

Principal Component Analysis (PCA) of the physiological dataset was conducted in R Statistical Software v.3.5.0 [35] using *FactoMineR* package v1.41 [51], *ggplot2* v3.0.0 [52] and *factoextra* v1.0.5 [53]. A PCA with non-standardized data was considered (variance-covariance matrix) due to the inherent data structure (linear dataset with variables measured on the same scale) [54]. The significance of each factor and the interaction was assessed using a non-parametric multivariate analysis of variance (NPMANOVA or perMANOVA; [55]) by implementing *adonis* function in *vegan* package v2.5–2 [56] using a Euclidean distance matrix (α = 0.05). When M×S interaction was detected (*P-*value < 0.2), suppression methods were compared separately at each sampling time. Analysis of similarities (ANOSIM) test was implemented for these comparisons (α = 0.05). Values of R-statistic close to 0.75 or higher in ANOSIM test were considered as completely separated groups, higher than 0.5 as separated but overlapping and lower than 0.25 as barely separable [54]. Homogeneity of multivariate dispersions for the eight treatments was verified with a permutation test (α = 0.05; 999 permutations) using *betadisper* function in *vegan* package.

To assess overall variation in community structure among treatments (i.e. beta-diversity), rarefied data from metabarcoding profiles were subjected to a multivariate analysis using a dissimilarity measure (Bray-Curtis) and a phylogenetic distance metric (Generalized UniFrac). Ordination was performed using non-metric multidimensional scaling (NMDS) in *vegan* package v2.5–2 [56] from R Statistical Software v.3.5.0 [35]. A stress value in NMDS around 0.1 or lower was considered a good agreement between the rank of similarities in the ordination and the rank from the original dissimilarity/distance matrix. For calculation of Generalized UniFrac distances, a phylogenetic tree was first estimated using *ape* package v5.1 [57] and *phangorn* package v2.4.0 [58]. Sequences were aligned and a neighbor-joining tree calculated. The likelihood of this tree was computed (*pml* function) and then optimized (*optim*.*pml*) using GTR model of nucleotide evolution. The phylogenetic tree was used in *GUniFrac* package v1.1 [59] to calculate distances (α-parameter = 0.5). To study the effects of M and S, a NPMANOVA (α = 0.05) using distance matrices [55] was used through *adonis* function (10^3^ permutations) in *vegan* package. The homogeneity of multivariate dispersions among treatments was visualized using *betadisper* function and a permutation test (α = 0.05; 999 permutations). To compare suppression methods at each sampling time ANOSIM test was used, with the same considerations that were mentioned before about R-statistic value. An indirect-gradient analysis approach was used for fitting environmental class variables (M = “glyphosate”, “mowing”; S = “4 days” “26 days”) onto ordination and goodness of fit assessed using the *envfit* function in *vegan* package.

The relative abundance (RA) of the different taxa in metabarcoding profiles, under mowing (RA_C_) and glyphosate treatments (RA_G_), were compared using STAMP Software [39] and White’s non-parametric t-test [60] with 10^3^ permutations at 5% significance level. Two filters of effect size [61] were applied to take into account those differences which could be biologically relevant even when a *P*-value > 0.05 was observed: the difference of proportions (DP, |DP| > 1.5%) with DP = RA_G_—RA_C_ and the ratio of proportions (RP, RP > 1.5). The RP corresponds to the ratio of the higher RA over the lower RA (i.e., RP > 1). The logical operator “OR” was used (features failing either condition were filtered). The cut-off values for filters (RP = 1.5, |DP| = 1.5%) were selected based on a trade-off between the default values in the software (RP = 2, |DP| = 1%) and the astringency of filters (harsh filtering conditions observed with values equal or higher than 2). Heatmaps were also constructed in STAMP Software using Unweighted Pair Group Method with Arithmetic Mean (UPGMA) algorithm and dendrogram similarity threshold of 0.75.

## Results

### Physiological analysis

#### Catabolic profiles

Higher SIR responses were observed in the rhizosphere of glyphosate-treated plants for all the tested substrates (S1a Fig), with increasing relative differences during the 26-day period (S1b Fig). Most significant differences were detected at 26 days (S1a Fig). At this sampling time, all substrates showed a significantly higher response under glyphosate treatment (*P* < 0.05). In addition, distinctive patterns were observed at the other sampling times for amino acids (phenylalanine, asparagine and sarcosine) and for Tween 20. Phenylalanine and asparagine showed a significantly higher response for the glyphosate treatment at 4 days. A similar but non-significant trend was observed for sarcosine at 4 days (*P* = 0.053) and at 10 days (*P* = 0.056). For Tween 20 a higher response was detected for the glyphosate treatment at 4 days and 17 days (*P* < 0.05; S1 Fig). The carbon availability index (CAI) was significantly lower at 10 days relative to 4 days for both suppression methods (*P* < 0.05). However, no differences were observed by the last sampling time (S2 Fig).

Principal component analysis of the physiological dataset indicated a clear separation in the ordination of microbial communities from glyphosate-treated plants and cut plants at 26 days and also a similar but weaker trend was evidenced at 4 days (S3 Fig). The separation was observed along the principal component 1 (Dim1) which explained a high percentage (88.56%) of the total variance. The contributions of the different variables to this principal component are indicated in S2 Table. Statistical analysis of the physiological dataset through NPMANOVA indicated a statistically significant interaction (*P* = 0.037), thus, comparisons between suppression methods were conducted separately at each sampling time (ANOSIM test, S3 Table). The separation observed in PCA (S3 Fig) was confirmed in ANOSIM test which indicated a statistically significant separation between suppression methods only at the last sampling time (*P* < 0.05, S3 Table).

**Fig 1:**
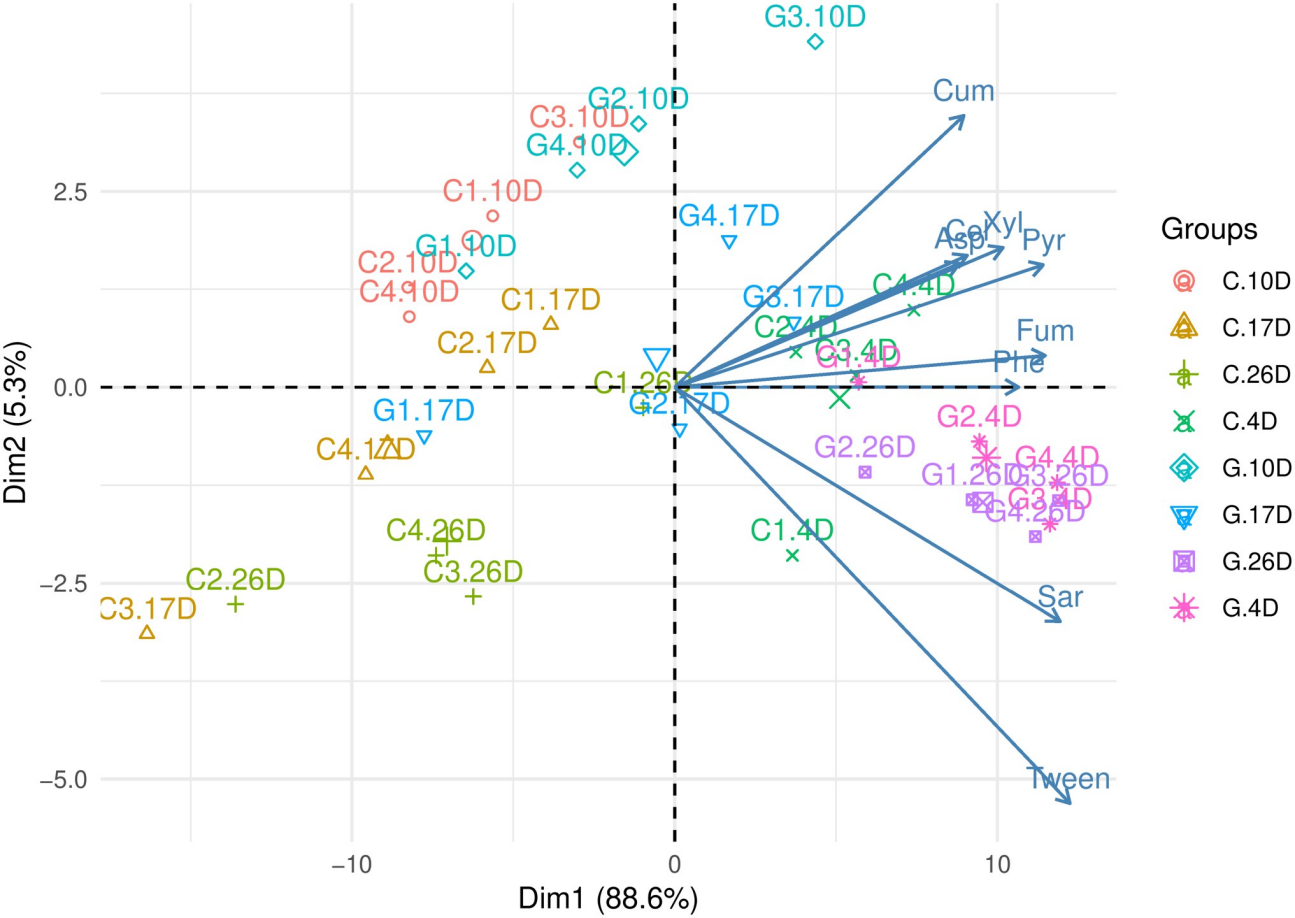
Principal component analysis (PCA) biplot of catabolic profiles. Variables are indicated in blue vectors while observations are represented by different colours and symbols according to the treatment (legend “*Groups*”). Letter C indicates mowing suppression and letter G glyphosate suppression. The number after these letters indicates the replicate, while the number after the point indicates the sampling time (.4D: 4 days; .10D: 10 days; .17D: 17 days; .26D: 26 days).

The representation of variables and observations in the space defined by Dim1 and Dim2 is observed in Fig 1. This biplot clearly showed the contribution of sarcosine and Tween 20 to the separation of microbial communities of glyphosate-treated plants (sampled at 4 and 26 days) from those of cut plants. Also, a clear trend was observed: the catabolic responses of microbial communities in the rhizosphere of cut plants decreased from the initial sampling time (4 days) to the last sampling time (26 days). Instead, in the rhizosphere of glyphosate-treated plants a decrease was also observed toward 17 days but the response at 26 days was similar to the response observed at 4 days.

The ratios of responses to root exudates (R_Ex_ = response to Ex_G_ / response to Ex_c_) are shown in S4 Fig. At 4 days, a R_Ex_ < 1 ratio was observed (i.e., lower response to Ex_G_) while at day 10 and 17 a R_Ex_ > 1 was detected. Significantly lower R_Ex_ values were observed for microbial communities of glyphosate-treated plants at the latest sampling times (17 and 26 days, *P* < 0.05).

#### Functional diversity

Catabolic diversity was assessed considering evenness (*E*) and Shannon diversity index (*H’*). As M×S interaction was observed (S4 Table), comparisons were conducted separately at each sampling time (Table 2). A higher value of *E* and *H’* was detected for rhizospheric microbial communities of glyphosate-treated plants taken at 26 days (*P* < 0.05). No significant differences were detected at 4, 10 and 17 days (Table 2). When comparing responses among different sampling times, a significant reduction in catabolic indices was detected at 10 days compared to 4 days, regardless of the suppression method considered (Table 2).

**Table 2:** Catabolic diversity of microbial communities. Evenness (*E*) and Shannon index (*H’*) values are indicated for each treatment. Values between brackets indicate the standard error of the mean (n = 4). Upper case letters indicate statistically significant differences between suppression methods at each sampling time. Lower case letters indicate statistically significant differences among sampling times within a suppression method (*P* < 0.05, Tukey’s HSD test).

Sampling time (S)	Shannon diversity index (*H’*)		Catabolic Evenness (*E*)	
	Mowing (C)	Glyphosate (G)	Mowing (C)	Glyphosate (G)
4 days	3.16 (9.9×10^−4^) ^**Aa**^	3.16 (2×10^−3^) ^**Aa**^	8.93 (1.1×10^−2^) ^**Aa**^	8.91 (2.5×10^−2^) ^**Aa**^
10 days	3.08 (5.7×10^−3^) ^**Ab**^	3.1 (7.4×10^−3^) ^**Ab**^	8.05 (6.2×10^−2^) ^**Ab**^	8.29 (7.6×10^−2^) ^**Ab**^
17 days	3.12 (1.4×10^−2^) ^**Ac**^	3.14 (5.4×10^−3^) ^**Aa**^	8.44 (0.14) ^**Ac**^	8.7 (6.4×10^−2^) ^**Aa**^
26 days	3.13 (9.3×10^−3^) ^**Ac**^	3.17 (9.6×10^−4^) ^**Ba**^	8.53 (9.1×10^−2^) ^**Ac**^	8.97 (1.1×10^−2^) ^**Ba**^

### Metabarcoding

As mentioned previously, samples from the first and last sampling time (4 days and 26 days) were analyzed for each suppression method by 454 pyrosequencing of 16S rRNA gene amplicons. A total of 46,239 pyrotags were obtained with an average length of 288 nucleotides after barcode and primer removal, including those of Bacteria and Archaea. The dataset has been deposited in Sequence Read Archive (SRA) repository under the accession PRJNA508531. The number of observed OTUs for the total dataset (12 samples) was 2063 (Bacteria+Archaea). Among them, 2047 were taxonomically assigned to Bacteria (99.2%), 15 to Archaea (0.73%) and 1 OTU (0.048%) could not be assigned to any of them. The number of sequences for each sample is indicated in S5 Table (non-rarefied data) and rarefaction curves in S5 Fig.

#### Alpha-diversity

Richness, evenness and diversity indices are indicated in Table 3. Statistical analysis through two-way ANOVA showed no significant interactions (*P* > 0.05, S6 Table) and, consequently, main effects were considered. According to the results, microbial communities in the rhizosphere of glyphosate-treated and cut plants showed similar alpha-diversity metrics, with no significant effect of suppression method (*P* > 0.05, S6 Table). Similarly, no significant effect of sampling time was detected.

**Table 3:** Alpha-diversity metrics from the metabarcoding dataset. Mean values are indicated for each treatment. Values between brackets indicate the standard error of the mean (n = 3). S’: observed richness; Chao-1: Chao-1 index (estimated richness); *H’*: Shannon index; 1/D: reciprocal of Simpson index; J’: Pielou’s index; *R*_*1*:*2*_: Hill-ratio.

Alpha-diversity metrics	Mowing		Glyphosate	
	4 days	26 days	4 days	26 days
**S’**	649.33 (27.09)	651 (50.36)	651.67 (31.35)	710.67 (20.34)
**Chao-1**	1101.45 (25.76)	1054.78 (93.11)	1154.05 (137.86)	1206.62 (65.99)
***H’***	5.74 (0.063)	5.78 (0.2)	5.73 (0.11)	5.97 (0.064)
**1/D**	122.54 (5.85)	152.91 (39.51)	124.52 (32.39)	180.08 (16.77)
**J'**	0.89 (0.0041)	0.89 (0.02)	0.88 (0.013)	0.91 (0.0066)
***R***_**1:2**_	2.57 (0.21)	2.34 (0.27)	2.73 (0.47)	2.19 (0.092)

#### Beta-diversity

**Fig 2:**
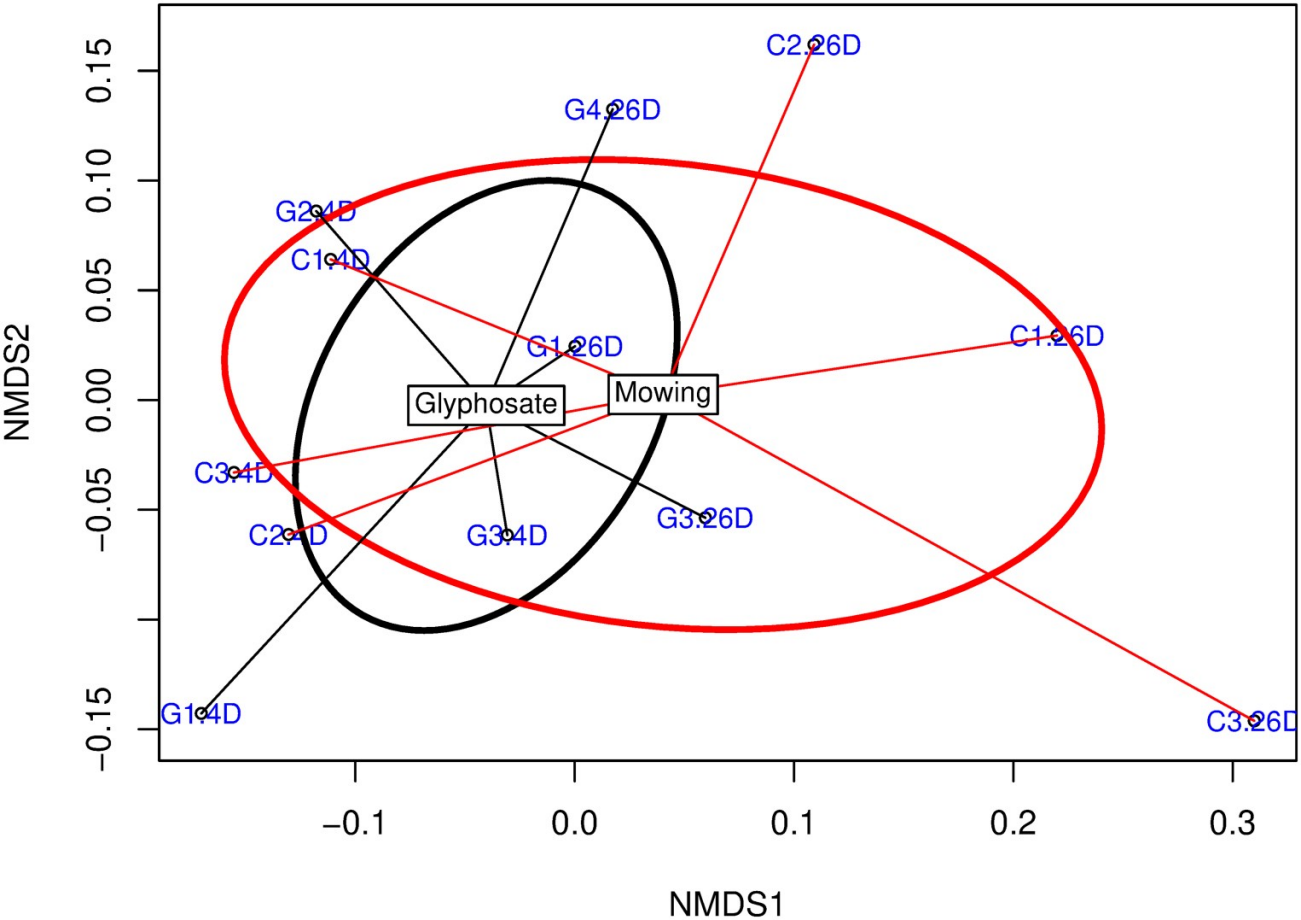
Multivariate analysis of the metabarcoding dataset in the rhizosphere of *Avena sativa*. Letter C indicates mowing suppression and letter G desiccation with glyphosate. The number after the letter refers to the replicate while the number after the point indicates the sampling time (.4D: 4 days; .26D: 26 days). Centroids are indicated in black boxes. Standard deviation is shown by red (C) or black (G) ellipses. Ordination was conducted using non-metric multidimensional scaling (NMDS) and Generalized UniFrac distance metric. Stress-value = 0.082.

The results of multivariate analysis of microbial communities through NMDS indicated consistent results for Bray-Curtis dissimilarity (S6 Fig) and generalized UniFrac distance (Fig 2). A separation was observed between samples taken at 4 and 26 days. However, bacterial communities in the rhizosphere of glyphosate-treated plants (black, Fig 2 and S6 Fig) did not show a clear separation from communities in the rhizosphere of cut plants (red). As indicated in S7 Table, this trend was supported by the results of goodness of fit of class variables M and S fitted to the NMDS ordination space (statistical significance was detected only for S).

According to the results of NPMANOVA test, an interaction (M×S) was observed (*P* = 0.098 for Bray-Curtis distance and *P* = 0.097 for Generalized UniFrac). Thus, suppression methods were compared separately at each sampling time (Table 4). No clear separation between microbial communities of cut and glyphosate-treated plants was observed at 4 or 26 days according to R-statistic values in ANOSIM test.

**Table 4:** Statistical comparison of the metabarcoding dataset between suppression methods at each sampling time. The result of analysis of similarities test (ANOSIM) is indicated.

Comparison	ANOSIM (Bray-Curtis)		ANOSIM (Generalized UniFrac)	
	*R-statistic*	Significance	*R-statistic*	Significance
**Mowing *vs* Glyphosate** **(4 days)**	0.037	0.3	0	0.5
**Mowing *vs* Glyphosate** **(26 days)**	0.3	0.1	0.48	0.1

A second pattern, similar to the one mentioned in physiological analysis (Fig 1), was detected in the ordinations of Fig 2 and S6 Fig: microbial communities from glyphosate-treated plants at 26 days (G.26D) were located in close proximity to communities sampled at 4 days (C.4D and G.4D) while this result was not detected for microbial communities of cut plants (C.26D).

#### Comparative analysis of bacterial taxa

**Fig 3:**
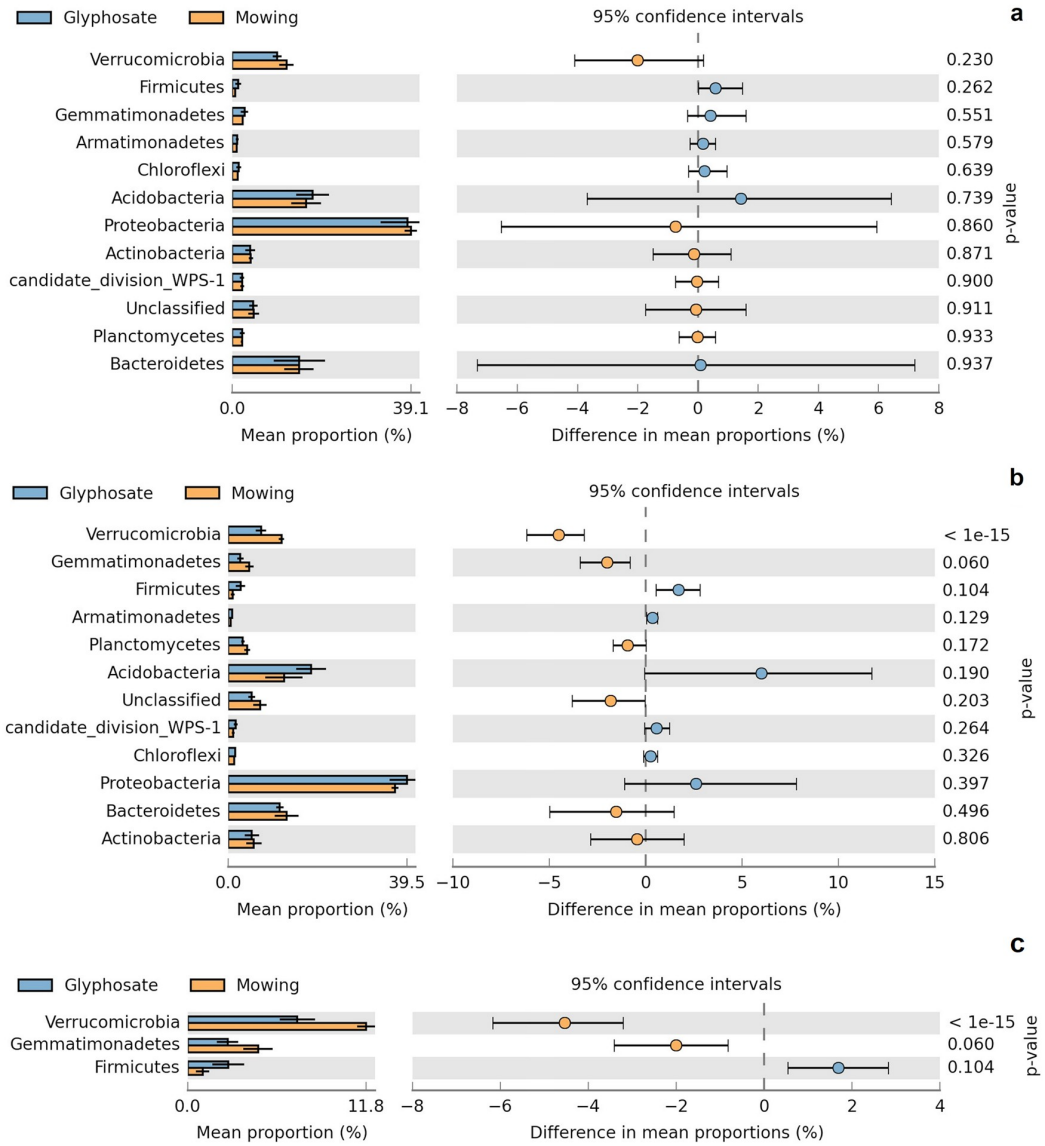
Comparison of mean relative abundance between suppression methods at phylum level. Panel a: 4 days (without effect size filters), Panel b: 26 days (without effect size filters). Panel c: 26 days (with effect size filters: |DP| > 1.5%; RP > 1.5). At 4 days after suppression, differences in mean proportions (DP) and in the ratio of proportions (RP) did not pass the filtering step. White’s non-parametric t-test was used for statistical analysis (α = 0.05). Only those categories with a minimum relative abundance of 0.25% in each sample are shown. Category “*Unclassified*” refers to bacterial sequences without taxonomic affiliation. Confidence intervals and *P*-values are indicated in each case.

As a first approach, graphical visualization of relative abundances of the several phyla detected at each sampling time indicated a similar distribution for mowing and glyphosate (S7 Fig). A clear dominance of *Proteobacteria* was detected relative to other phyla, followed by *Acidobacteria* and *Bacteroidetes*. The comparative statistical analysis at phylum level is indicated in Fig 3. According to these results, neither significant nor biologically relevant differences (*P* > 0.05; |DP| < 1.5%, RP < 1.5) were observed between suppression methods at 4 days (Fig 3a). Instead, at 26 days *Verrucomicrobia* showed a statistically significant difference (*P* < 0.05; |DP| = 4.53%; RP = 1.63) with a lower value of relative abundance in the rhizosphere of glyphosate suppressed plants (Fig 3b). Effect size statistics filters (|DP| > 1.5%, RP > 1.5) indicate this difference could be biologically relevant (Fig 3c), as will be further discussed. The comparison of sampling times indicated a reduction in the abundance of *Verrucomicrobia* from 4 days to 26 days under glyphosate suppression but not under mowing suppression (S8 Fig).

**Fig 4:**
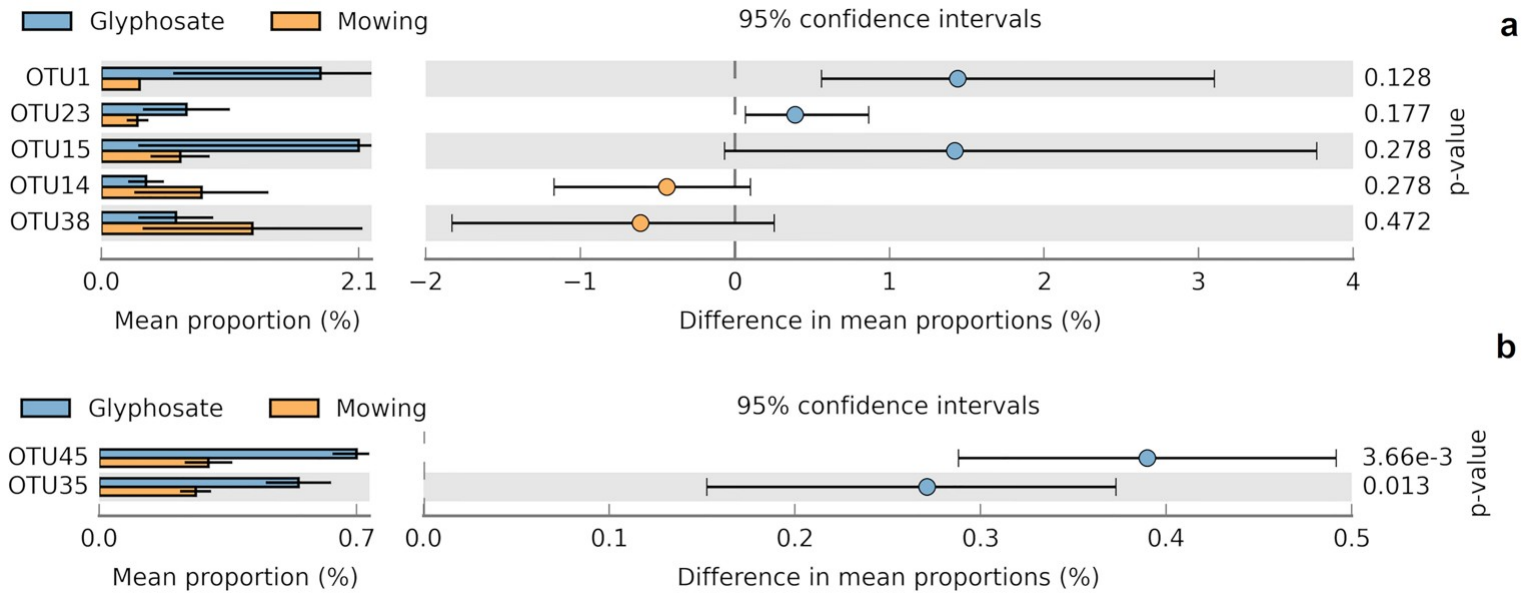
Comparison of mean relative abundance between suppression methods at OTU level (with effect size filters). Panel a: 4 days, Panel b: 26 days. White’s non-parametric t-test was used for statistical analysis (α = 0.05). Only those categories with a minimum relative abundance of 0.2% in each sample are shown. Confidence intervals and *P*-values are indicated in each case. Effect size filter: RP > 2.

Biologically relevant differences were also detected at lower taxonomic levels. Comparative analysis at genus level indicated a significant difference in *Rhodoplanes* at 4 days (S9 Fig), with a higher abundance in the rhizosphere of glyphosate-treated plants (|DP| = 0.29%, RP = 1.63). Although no significance was detected for *Hydrogenophaga*, the result followed the same trend than *Rhodoplanes* and could be biologically relevant as reflected by the highest effect size statistics (|DP| = 1.64%, RP = 3.62) (S9b Fig). Biologically relevant results were also observed for *Devosia* (|DP| = 0.86%, RP = 2.21) and *Sphingomonas* (|DP| = 0.93%, RP = 1.51) but an opposite trend was observed in these cases, with a lower relative abundance under glyphosate suppression (S9b Fig). The results mentioned before were not consistently observed at 26 days (S9c–S9d Fig), with the exception of *Devosia* which showed a lower relative abundance again under glyphosate suppression (|DP| = 0.93%, RP = 1.51). At this sampling time, a significantly higher relative abundance was only detected for *Mesorhizobium* (|DP| = 0.35%; RP = 1.95) in the rhizosphere of glyphosate-treated plants and for *Gaiella* in the rhizosphere of cut plants (|DP| = 0.25%; RP = 1.68). Comparative analysis at OTU level indicated biologically relevant differences in five OTUs at 4 days (Fig 4a) and in two OTUs at 26 days (Fig 4b). Five of them were identified up to genus level as indicated in S8 Table.

A cluster analysis was conducted with most abundant OTUs in *Betaproteobacteri*a, which comprises several glyphosate degraders. As shown in S10 Fig, a neat separation between suppression methods was only detectable at 4 days post-treatment.

### Quantitative PCR of indicator genes

The abundance of total bacteria, Archaea, *Actinobacteria*, AOB and AOA were estimated through qPCR of the respective indicator genes. The relationship between the copy number and *Ct* values were described by the equations specified in S9 Table. Efficiencies are also indicated in S9 Table.

The results of two-way ANOVA of the different indicator genes are indicated in S10 Table. For *amoA*_AOB_ no significant M×S interaction was detected and, thus, main effects were considered. Statistical significance was observed for the main effect of suppression method (*P* < 0.05, S10 Table). The copy number of *amoA* was 1.68 fold higher in the rhizosphere of cut plants than in glyphosate-treated plants (Table 5).

**Table 5:** Copy number of indicator genes from different microbial groups in the rhizosphere of glyphosate-treated or cut plants. Mean values were calculated through all sampling times (no interaction detected, n = 16). Values between brackets indicate the standard error of the mean. Only those microbial groups in which no interaction was observed are shown. Different letters indicate statistically significant differences (*P* < 0.05).

Copy number of indicator gene μg^-1^ DNA				
	AOB	*Actinobacteria*	Total bacteria	Archaea
**Mowing**	1.61×10^5^ (1.66×10^4^) ^**b**^	2.85×10^7^ (3.26×10^6^) ^**a**^	2.38×10^9^ (2.16×10^8^) ^**a**^	6.76 ×10^6^ (6.53×10^5^) ^**a**^
**Glyphosate**	9.56×10^4^ (1.85×10^4^) ^**a**^	2.97×10^7^ (2.66×10^6^) ^**a**^	2.89×10^9^ (2.95×10^8^) ^**a**^	7.81 ×10^6^ (6.34×10^5^) ^**a**^

For AOA, glyphosate and mowing treatments were compared within each sampling time as M×S interaction was observed (*P* = 0.14). A statistically significant difference (*P* = 0.037) was detected only at 26 days: the abundance was 2.4 fold higher in the rhizosphere of cut plants (Table 6). As indicated in S1 File, *amoA*_AOA_ was detected in a higher abundance in the rhizosphere than *amoA*_AOB_, regardless of the suppression method or the sampling time considered. No significant differences (*P* > 0.05) were detected in AOA:AOB ratio between glyphosate (AOA:AOB = 50.89) and mowing (AOA:AOB = 28.12) suppression (S10 Table).

**Table 6:** Copy number of *amoA* gene of ammonia-oxidizing archaea (AOA) in the rhizosphere of glyphosate-treated or cut plants. Values between brackets indicate the standard error of the mean (n = 4). Different letters indicate statistically significant differences (*P* < 0.05, two-sample t-test).

Copy number of *amoA* μg^-1^ DNA				
	4 days	10 days	17 days	26 days
**Mowing**	2.04×10^6^ (3.82×10^4^) ^**a**^	3.95×10^6^ (7.71×10^5^) ^**a**^	5.87×10^6^ (1.96×10^6^) ^**a**^	3.57 ×10^6^ (8.69×10^5^) ^**b**^
**Glyphosate**	3.51×10^6^ (9.49×10^5^) ^**a**^	2.65×10^6^ (6.90×10^5^) ^**a**^	4.34×10^6^ (8×10^5^) ^**a**^	1.49 ×10^6^ (3.42×10^5^) ^**a**^

Statistical analysis for total bacteria, Archaea and *Actinobacteria* showed no interaction effects. Thus main effects were considered (Table 5 and S10 Table). Contrary to AOB, no significant effect of suppression method was detected for total bacteria, Archaea and *Actinobacteria*. Instead, a significant effect of sampling time (*P* < 0.05) was observed (S10 Table).

### Culture-independent analysis of IncP-1 plasmids

#### Quantitative PCR of IncP-1 plasmids

IncP-1 plasmids were below the detection limit or not detected in total community DNA by qPCR (S11 Table). A highly specific and sensitive method (PCR-Southern blot) was selected for detection of IncP-1 plasmids (next section).

#### Detection of IncP-1 plasmids by PCR-Southern blot

**Fig 5:**
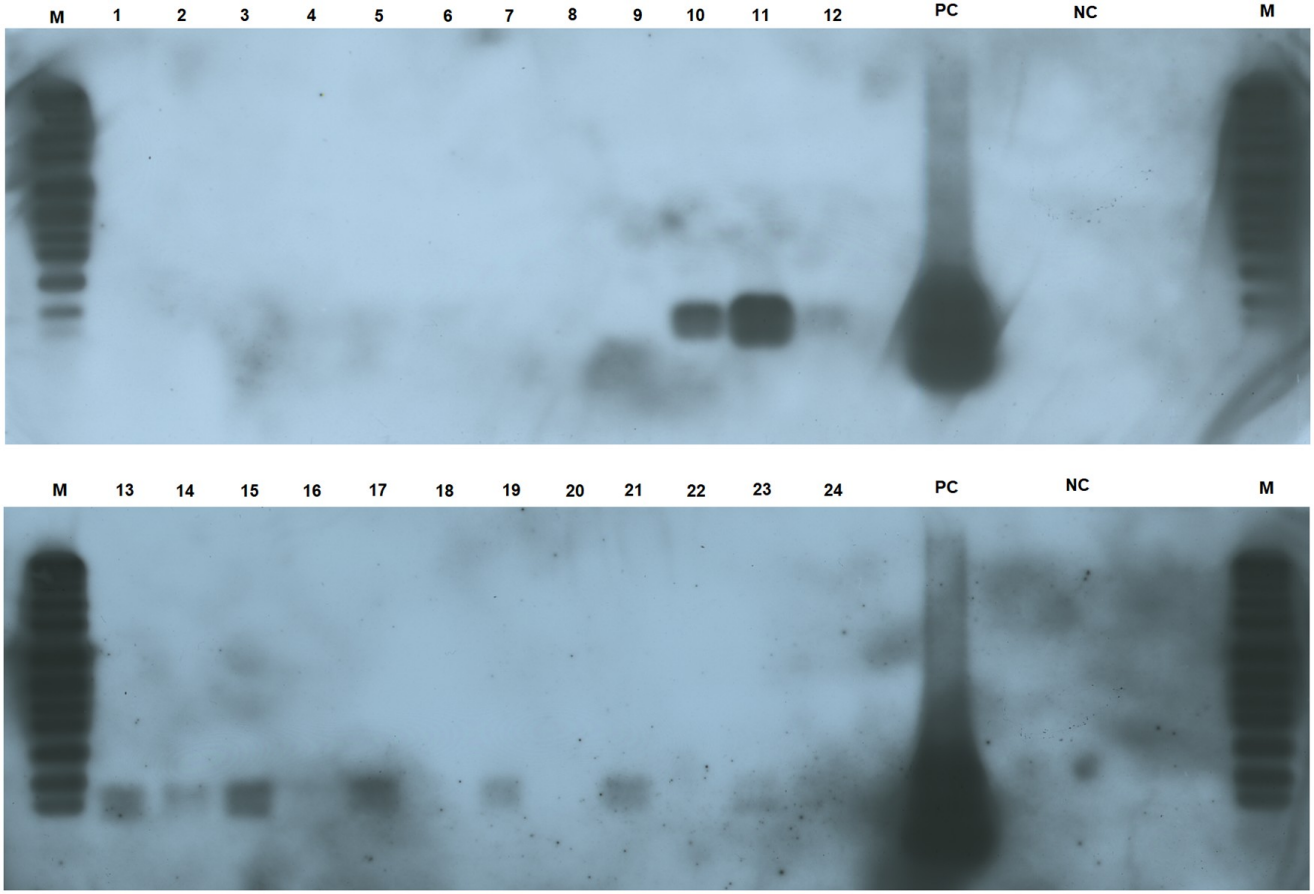
PCR-Southern blot using a probe for IncP-1β subgroup. Lanes: 1–3: Mowing/4 days; 4–6: Glyphosate/4 days; 7–9: Mowing/10 days; 10–12: Glyphosate/10 days; 13–15: Mowing/17 days; 16–18: Glyphosate/17 days; 19–21: Mowing/26 days; 22–24: Glyphosate/26 days; M = marker DIGVII; PC: positive control (amplification product of IncP-1β plasmid pR751); NC: negative control. Exposure time: 30 min. In those lanes in which no bands were detected, the same result was observed after a longer exposure time. Size of the smallest band of DIGVII marker: 359 bp.

The methodological approach of PCR followed by hybridization of amplicons with IncP-1 specific probes showed a high sensitivity and specificity in the detection of plasmids. When an IncP-1β specific probe was used, plasmids were not detected at 4 days and the most relevant observation was observed at 10 days. At this sampling time, plasmids were detected only in the rhizosphere of glyphosate-treated plants (Fig 5) but this difference reverted at 26 days after suppression.

**Fig 6:**
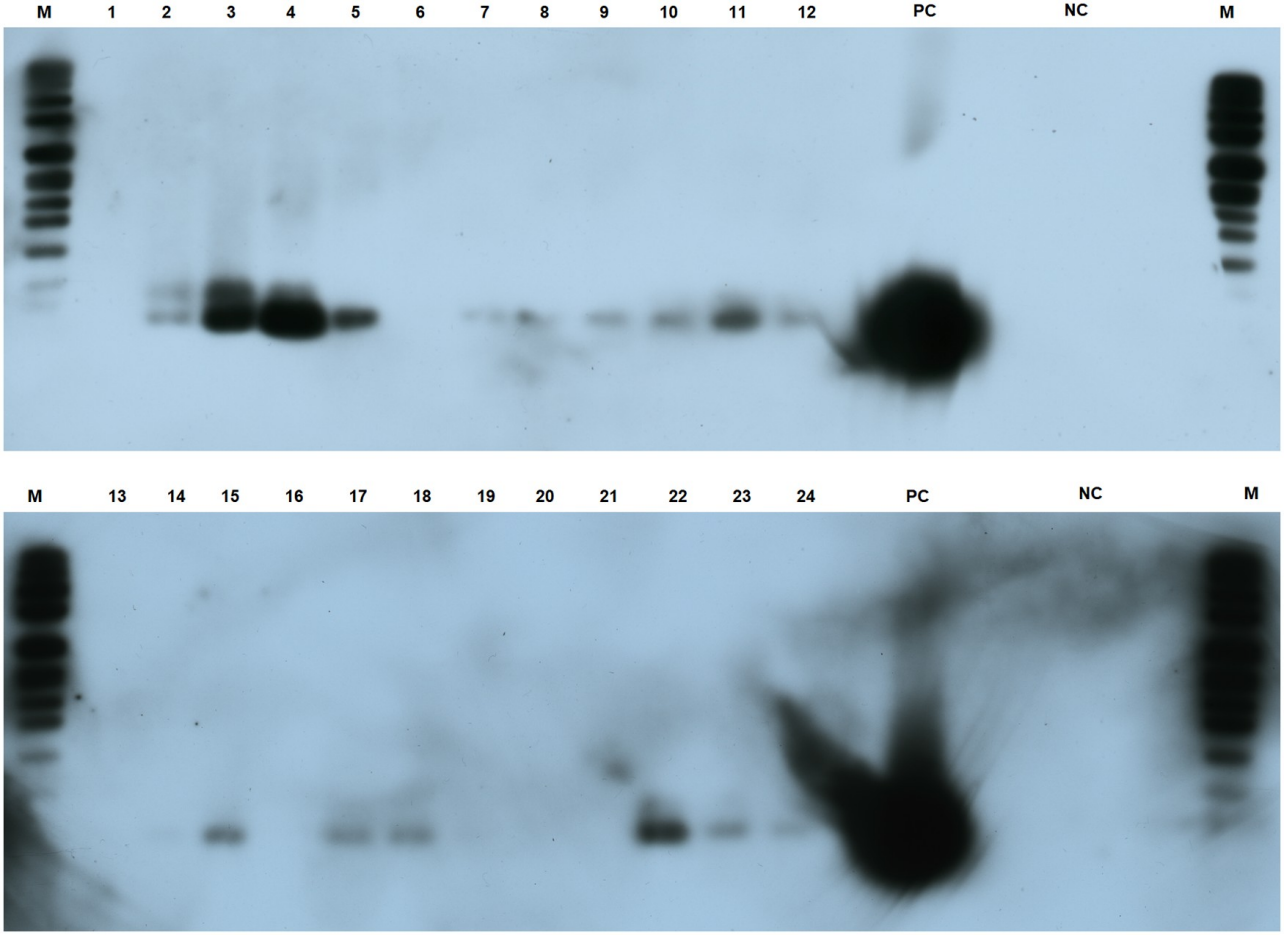
PCR-Southern blot using a probe for IncP-1ε subgroup. Lanes: 1–3: Mowing/4 days; 4–6: Glyphosate/4 days; 7–9: Mowing/10 days; 10–12: Glyphosate/10 days; 13–15: Mowing/17 days; 16–18: Glyphosate/17 days; 19–21: Mowing/26 days; 22–24: Glyphosate/26 days; M = marker DIGVII; PC: positive control (amplification product of IncP-1ε plasmid pKJK5); NC: negative control. Exposure time: 30 min. In those lanes in which no bands were detected, the same result was observed after a longer exposure time. Size of the smallest band of DIGVII marker: 359 bp.

Similarly to IncP-1β, a result of presence/absence of plasmids was detected for IncP-1ε. However, the result was observed at 26 days and not at 10 days. At the last sampling time, IncP-1ε plasmids were detected only in the rhizosphere of glyphosate-treated plants (Fig 6). In addition, this subgroup was observed from the beginning (4 days) for both suppression methods, contrary to IncP-1β subgroup.

## Discussion

The effects of desiccation of a widely cultivated CC (*A*. *sativa*) on rhizospheric microbial communities were studied in relation to suppression without herbicide (mowing) with the aim to assess which microbial groups could differ and the implications for soil processes. A ‘control’ treatment of intact plants was not included as it clearly does not represent a suitable non-glyphosate control from an agronomic point of view (i.e., the CC is necessarily suppressed by chemical or mechanical methods). To accomplish our objective, we used a holistic approach, evaluating the bacterial diversity (alpha/beta-diversity and taxonomic profiles), the abundance of ecologically relevant microbial groups, the structure of bacterial communities (*Betaproteobacteria*) and the functional diversity. Most importantly, this is the first time that the presence/absence of ecologically relevant BHR plasmids (IncP-1) was evaluated under GBH treatment. Results indicated that microbial communities are differentially influenced by suppression methods.

### Catabolic profiles and functional diversity

Statistical analysis of catabolic profiles indicated significant differences for the nine tested substrates at the last sampling time (26 days) and for amino acids at 4 days (S1 Fig). Considering that glyphosate has been reported to modify the quality and quantity of root exudates increasing the content of amino acids [6,9], a higher activity of microbial communities in the rhizosphere of glyphosate-treated plants could be expected (reflected in the higher SIR responses observed for amino acids at 4 days) (S1 Fig). The higher respiratory response could also be explained by the contribution to the rhizodeposits of C derived from turnover of dead root tissue, as glyphosate accelerates root biomass turnover enhancing carbon substrate availability [9]. This second explanation could better explain the stimulation of respiration observed at the last sampling time (26 days) due to a progressive decay of dead root tissue.

Principal component analysis (PCA) of physiological data indicated a neat separation between profiles from both suppression methods only at 26 days after treatment (S3 Fig), in agreement with the results observed for similar treatments in triticale rhizosphere [12]. The separation was observed along the principal component which explained the highest percentage of variance (Dim1). Sarcosine and Tween 20 showed the highest contribution to this component (S2 Table). The result observed for sarcosine is particularly relevant for microbial communities of glyphosate-treated plants considering that sarcosine is the main product of glyphosate degradation by C-P lyase pathway, in bacterial and fungal strains [62]. Thus, a higher response could be indicating a higher abundance of microorganisms capable of glyphosate degradation and consequently capable of sarcosine degradation. Similarly, the higher response in the rhizosphere of glyphosate-treated plants with Tween 20 could be related with a higher abundance of microorganisms adapted to a rapid utilization of C compounds (ethoxylated chains) derived from Tween degradation. This result should be further explored considering that ethoxylated compounds are commonly used surfactants in a wide range of pesticide formulations [63], including GBHs [64].

Catabolic diversity was analyzed through evenness and a diversity index (Table 2). Results indicated significantly higher values under glyphosate treatment only at 26 days, consistent with the ordination pattern observed in PCA (Fig 1) while Mijangos et al. [12] reported a higher *H’* value in triticale relative to cut plants at 15 days post-treatment and a lower value at 30 days.

A significant reduction was observed for functional diversity parameters for both glyphosate-treated and cut plants at 10 days relative to 4 days (Table 2). A decline in *E* parameter is normally observed in microbial communities under unfavorable conditions, particularly when the intensity of the perturbation (e.g. dry-rewetting cycles, heavy metal pollution) is high [30]. Microbial communities in the rhizosphere may have faced C limitation by 10 days (as reflected by lower values of carbon availability index, S2 Fig), induced by the suppression of the CC, which showed evident symptoms of desiccation at 10 days but not at 4 days. Our results are in line with those reported by Kremer et al. [6] who reported a level off trend in the rate of exudation of carbohydrates between 8 and 16 days post-treatment relative to the values observed at 2 and 4 days in a glyphosate sensitive soybean cultivar. The higher values of catabolic diversity and CAI at latest sampling times (Table 2 and S2 Fig) could be related with the exposure of communities to substrates released during turnover of root material, as explain earlier.

Regarding respiratory responses to root exudates we observed both R_Ex_ > 1 and R_Ex_ < 1 values (S4 Fig). The R_Ex_ > 1 indicates a stimulation of microbial activity by Ex_G_ exudate. Contrary, lower responses with Ex_G_ (R_Ex_ < 1) may be expected in microbial communities more adapted to the rapid assimilation of products from glyphosate degradation or root-derived compounds rather than respiration. At 17 and 26 days, a significantly lower R_Ex_ was observed in microbial communities of glyphosate-treated plants relative to microbial communities of cut plants. In addition, while R_Ex_ values higher than 1 were observed for microbial communities of cut plants at both sampling times, the R_Ex_ for glyphosate suppression was higher than 1 only at 17 days. R_Ex_ values higher or lower than 1 at different sampling times within a given suppression method might be attributed to: 1) physiological differences of microbial communities sampled at different sampling times; 2) differences in the root exudates collected at each sampling time; 3) both possibilities.

### DNA-based methods

Among the huge diversity of microorganisms in the rhizosphere, Bacteria comprises the most studied glyphosate-degraders [62]. Similarly, IncP-1 plasmids have been isolated and characterized from several bacterial taxa. Regarding nitrification, both bacteria (AOB) and archaea (AOA) are the main ammonia-oxidizers. Thus, we focused on DNA-based analysis of bacterial and archaeal taxa. However, it must be highlighted that fungi in the rhizosphere should be assessed in future studies, especially arbuscular mycorrhiza which have shown a reduction in spore viability and root colonization due to GBHs [65] and *Fusarium* spp. for which a stimulation has been reported [66].

#### Metabarcoding

Only sequences from Bacteria were considered in 16S amplicon sequencing analysis. The universal pair of primers for prokaryotes used in this study (515F-806R) is not completely appropriate to study Archaea due to the limited ability to capture the diversity of these microorganisms [67]. Other studies with primers 515F-806R have also been limited to Bacteria [68].

No significant differences were detected for alpha-diversity metrics between glyphosate and mowing at 4 days or at 26 days after treatment (Table 3 and S6 Table). Moreover, both diversity indices (*H’* and 1/D) with different sensitivity to more rare (*H’*) and dominant (1/D) OTUs did not show a significant treatment effect. These results are consistent with those reported by Newman et al. [69] in a metabarcoding study in the rhizosphere of soybean and maize GR genotypes after glyphosate treatment. Similarly to alpha-diversity results, no significant effect of the suppression method was detected in beta-diversity analysis (S7 Table) with a phylogenetic distance metric (Generalized UniFrac, Fig 2) or a dissimilarity measure (Bray-Curtis) (S6 Fig). Differences between mowing and glyphosate treatments were only detected when considering the relative abundance of taxa at different taxonomic levels (Figs 3 and 4, S9 Fig), or the functional diversity (Table 2). In general, variation in microbial community structure was more related with time-dependent effects than with the suppression method applied (S7 Table). Newman et al. [69] did not observe overall effects of a GBH on bacterial diversity in the rhizosphere of GR soybean and maize through metabarcoding. They concluded that the response of microbial diversity to herbicide application should be examined at a higher resolution level both taxonomically (relative abundance of specific taxa) as well as functionally rather than focusing only on net diversity responses. The same conclusion can be established based on our results. Some authors have indicated that the more commonly used community indices are insensitive to biological changes in natural communities and that erroneous conclusions can be established by using a single index [70]. Methods associated with the calculation of a diversity index or similarity indices, exclusively, are limited by this insensitivity and might be complemented by additional methods [70] or by additional analyses with the same dataset (as done in this study) in order to confirm or exclude changes in microbial communities.

Results of metabarcoding for *Actinobacteria* indicated no significant differences between suppression methods (consistently with qPCR). Similarly, no significant differences were detected for other relevant phyla in 16S amplicon sequencing profiles at 4 days (Fig 3a). However, a biologically relevant difference was observed for *Verrucomicrobia* at 26 days (Fig 3b). The oligotrophic life history strategy of *Verrucomicrobia* [71] and the higher exudation of amino acids and carbohydrates in the roots of glyphosate-treated plants [6] could probably explain its lower relative abundance compared to cut plants.

*Proteobacteria* showed a clear dominance in the rhizosphere of *A*. *sativa*, in comparison with other phyla and in agreement with the results observed in the rhizosphere of *A*. *fatua* [72]. Our results are also consistent with those reported by Vandenkoornhuyse et al. [73] who demonstrated that *Proteobacteria*, in contrast to *Actinobacteria* and *Acidobacteria*, are the most active bacteria assimilating root exudates.

When exploring lower taxonomic levels, a higher number of biologically relevant differences were observed (Fig 4 and S9 Fig). Although no statistical significance was observed for *Hydrogenophaga* and *Curvibacter* (*Burkholderiales*), they showed the highest values of effect size statistics, with higher relative abundance in the rhizosphere of glyphosate-treated plants at 4 days after treatment (S9 Fig). The result observed for OTU1 (*Hydrogenophaga* sp.) (Fig 4, S8 Table) is in agreement with the trend observed at genus level. *Hydrogenophaga* and *Curvibacter* belong to *Betaproteobacteria*, which showed a separation in cluster analysis at this sampling time (S10 Fig). These results are also consistent with Lancaster et al. [74] who reported an increase in the abundance of *Betaproteobacteria* after repeated applications of a GBH and with the reported ability of members of this taxon to degrade pesticides, including glyphosate degraders in *Burkholderiales*. Conversely, for other taxa we observed differences by the last sampling time. One the most remarkable results from metabarcoding was the significantly higher abundance of the genus *Mesorhizobium* (S9 Fig) and of OTU35 (Fig 4, S8 Table) in the rhizosphere of glyphosate-treated plants at 26 days. A BLASTn search in Greengenes database with OTU35 sequence revealed 99.6% identity (E-value = 1e-115, Score = 416 bits) with *Mesorhizobium loti* NGT514 (accession number AB289614.1 in GenBank). We propose that *M*. *loti* is influenced by suppression with GBHs due to its remarkable redundancy of genes involved in the catabolism of phosphonates (*phn* genes) encoding C-P lyase pathway (three *phn* loci). Although the ability to degrade glyphosate through C-P lyase activity is currently unknown in *M*. *loti*, the higher abundance of the genus *Mesorhizobium* and of OTU35 in the rhizosphere of glyphosate-treated plants, the reported exudation of glyphosate in different crops [5,6,8] and the presence of a complete set of genes for the catabolism of alkyl and aminoalkylphosphonates in *M*. *loti* [62] seem to support this metabolic ability.

#### Abundance of different microbial groups

The lower number of copies of *amoA*_AOB_ in the rhizosphere of glyphosate-treated plants (Table 5) is in agreement with the widely recognized sensitivity of AOB to different factors including pesticides like dazomet and atrazine [75,76]. Also, a significant inhibition of gross nitrification rate and *amoA* abundance (AOB and AOA) has been recently reported for a GBH in bulk soil [76]. The lower abundance could also be explained by low oxygen levels in the rhizosphere of glyphosate-treated plants (inhibitory levels) due to higher respiration responses of competing aerobic microorganisms triggered by an increased rhizodeposition of carbohydrates and amino acids [6].

Analysis of AOA qPCR data indicated a significantly lower abundance at 26 days in the rhizosphere of glyphosate-treated plants (Table 6). Similarly, previous studies on bulk soil have reported sensitivity of this nitrifying group to GBHs [76]. The lower abundance of *amoA* gene (for both AOA and AOB) in the rhizosphere of glyphosate-treated plants is a particularly relevant result considering the key ecological role of these microorganisms in N cycle in agricultural soils [77]. A lower abundance of AOA and AOB populations in remaining roots of *A*. *sativa* after glyphosate suppression could result in a lower nitrification activity and higher N-NH_4_^+^ availability for the following crop, particularly for AOB whose abundance is positively correlated with nitrification potential [78] and nitrate levels [47].

For total bacteria and *Actinobacteria*, the lack of significant differences in the abundance between suppression methods (S10 Table) was consistent with the results obtained by metabarcoding at phylum level (Fig 3). Other studies indicate that the relative abundance of this phylum is inhibited after treatments with GBHs in maize rhizosphere [79]. More studies are necessary to address potential effects on *Actinobacteria*. Indeed, we detected a lower abundance of the genus *Gaiella* (*Actinobacteria*) for glyphosate suppression (S9 Fig). A negative correlation has been recently observed between *Gaiellaceae* and the C:N ratio [80], which is probably increased by the rhizodeposition of carbohydrates after GBH treatment.

Archaeal 16S rRNA abundance was also unresponsive to the suppression method, in contrast to archaeal *amoA* abundance. Recently, Jenkins et al. [81] reported that both genes showed different response patterns to glyphosate in maize bulk soil and in the rhizosphere.

#### Analysis of mobile genetic elements in total community DNA

Horizontal gene transfer mediated by mobile genetic elements is a major force in the adaptation and diversification of bacteria and also in the robustness of microbial community response under changing conditions or new challenges and opportunities [14]. Even though quantification of IncP-1 plasmids was not successful, in spite of the optimized qPCR protocol [48], the combination of PCR of *trfA* gene and hybridization allowed a high sensitivity and specificity towards detection of different subgroups of IncP-1 plasmids with low abundance in the metagenomic DNA.

At 10 days after suppression, plasmids from IncP-1β subgroup were exclusively detected in the rhizosphere of glyphosate-treated plants (Fig 5). The same trend was observed for IncP-1ε at a different sampling time (26 days) (Fig 6). Different timing for the detection of IncP-1ε and β subgroups could be related with the specific functions encoded by each subgroup and the advantages conferred to the hosts. Specifically, increases in the relative abundance of IncP-1β plasmids have been observed over an agricultural season (March to September) in biopurification systems in which increasing concentrations of several pesticides were detected (e.g. diuron, flufenacete, metribuzine), while this increase was not observed for IncP-1ε [82]. Many authors have characterized IncP-1β plasmids harboring catabolic genes for different compounds (e.g. 2,4-D and atrazine) [83,84]. The detection of IncP-1β plasmids at 10 days may be linked to the degradation of exuded glyphosate, indeed, the main degradative pathways for phosphonates (C-P lyase and phosphonatase dependent pathway) have been subject to extensive lateral gene transfer and *phn* gene clusters have been found on plasmids [85]. It is known that the influence of selective pressure is a key environmental factor that can stimulate gene transfer processes [13] and studies of glyphosate exudation in soybean have shown the highest exudation rate in the first 12 days [6]. The horizontal transfer of *phn* genes from a strain harboring this type of plasmid genes (e.g. *M*. *loti*) to an IncP-1β plasmid in the first days after GBH treatment could explain our observation. However, more studies are necessary to test this putative explanation.

The detection of IncP-1ε plasmids in the rhizosphere of glyphosate-treated plants at 26 days (Fig 6) is particularly relevant considering that this subgroup has been recognized as an important vector for dispersion of antibiotic resistance genes in agro-ecosystems [86]. We suggest that the higher abundance of IncP-1ε plasmids could be related to the bacterial community changes in response to a higher rhizodeposition of carbohydrates and amino acids stimulated by glyphosate [6] and with the impairment of root growth [10]. A higher rhizodeposition would generate a higher availability of substrates, which could further explain a high cell density and a high metabolic activity of members involved in this energetically demanding process [13]. Both conditions would be favored by increased rhizodeposition, which also stimulates transconjugant proliferation [14]. Mølbak et al. [87] demonstrated that a higher exudation and a lower root growth rate are the two major factors that stimulate transfer frequencies of IncP-1 plasmids in barley rhizosphere. However, vertical transmission during proliferation of the plasmid containing population could also explain higher plasmid abundance [14]. Undoubtedly, more studies are necessary to disclose mechanisms underlying the result observed in Fig 6.

As far as we know, no similar studies of IncP-1 plasmids have been published after chemical suppression of CC, even though the exposure to increasing concentrations of pesticides has been associated with the enrichment of IncP-1 plasmids in microbial communities [15,82] and despite the risk of dissemination of antibiotic resistance genes in agro-ecosystems by IncP-1ε plasmids [86].

#### Conclusion and final remarks

The results of this study confirmed our original hypothesis: microbial communities in the rhizosphere of *A*. *sativa* are differentially influenced by the suppression method. The differences were observed at the physiological level (shifts in C use profiles), in the structure of microbial communities (shifts in specific taxa, such as *Betaproteobacteria*, *Verrucomicrobia*, *Mesorhizobium*, *Gaiella*), in the abundance of the main nitrifyers (AOA and AOB), and finally in the occurrence of IncP-1 plasmids. These parameters were more sensitive to explore the differential effects than alpha and beta diversity measurements.

The putative model presented in S11 Fig provides a link between our observations and literature information of glyphosate mode of action in other plant species, even when collected evidences in this study are not conclusive to demonstrate that glyphosate is solely responsible for the above mentioned differences. The higher content of carbohydrates and amino acids in rhizodeposits stimulated by glyphosate due to exudation and to turnover of necrotic root tissue could explain the higher microbial activity (higher response to C-substrates). In turn, this higher activity would create favorable conditions for horizontal gene transfer, explaining the detection of IncP-1ε plasmids at 26 days after suppression. In parallel, high microbial activity most probably decreased oxygen levels in the rhizosphere, partly explaining the lower abundance of AOB and AOA. Last, glyphosate exudation and its catabolism to sarcosine (by C-P lyases) could explain not only the higher response to sarcosine but also the higher relative abundance of *M*. *loti* harboring redundant *phn* loci for degradation of phosphonates through C-P lyase pathway.

This is the first study to depict the potential changes in ecologically relevant microbial processes in the rhizosphere of a cover crop under contrasting suppression managements. Nonetheless, several gaps in knowledge remain to be explored, particularly, whether nitrification rate is actually lower in glyphosate-treated plants and how IncP-1 plasmids may affect microbial community functions after GBH treatments. Future work should aim at capturing these plasmids based on their ability to mobilize IncQ plasmids and characterize their catabolic genes by full sequencing.

## Supporting information

S1 Text Amplification protocols. (PDF)

S2 Text Detection IncP-1 plasmids by Southern blot hybridization. (PDF)

S1 File Respiratory responses and copy numbers of indicator genes for each sample. (XLSX)

S1 Fig Catabolic profiles at each sampling time. Panel a = Absolute responses; Panel b: Relative differences. Error bars indicate the standard error of the mean (n = 4). The *P-*value is shown only for statistically significant differences (asterisk, two-sample t-test, *P* < 0.05). AUC: integrated area under the curve. NRFU: normalized relative fluorescence units.
(TIFF)

S2 Fig Carbon availability index (CAI) in rhizospheric soil for the different treatments. Upper case letters indicate statistically significant differences between suppression methods at each sampling time. Lower case letters indicate statistically significant differences among sampling times within a suppression method (*P* < 0.05, Tukey’s HSD test).
(TIFF)

S3 Fig Principal component analysis (PCA) of catabolic profiles. Confidence ellipses (95%) around replicates with a same treatment are indicated in purple. Treatments: 1–4: Mowing/4 days; 5–8: Glyphosate/4 days; 9–12: Mowing/10 days; 13–16: Glyphosate/10 days; 17–20: Mowing/17 days; 21–24: Glyphosate/17 days; 25–28: Mowing/26 days; 29–32: Glyphosate/26 days.
(TIFF)

S4 Fig Microbial respiration induced by root exudates. The ratio of responses to root exudates (R_Ex_ = Response to Ex_G_ / Response to Ex_C_) is shown for microbial communities of cut plants and glyphosate-treated plants. Error bars indicate the standard error of the mean (n = 4). Asterisks indicate significant differences according to two-sample t-test (*P* < 0.05), except for data at 26 days (Wilcoxon test, *P* < 0.05). Colours indicate that root exudates tested at 4, 10, 17 and 26 days were collected at the corresponding times, thus, no comparisons of responses were made among sampling times.
(TIFF)

S5 Fig Rarefaction curves of the different samples analyzed through metabarcoding. The number of operative taxonomic units (OTUs) of Bacteria is indicated for an increasing sampling effort (“sample size”).
(TIFF)

S6 Fig Multivariate analysis of the metabarcoding dataset in the rhizosphere of *Avena sativa* L. through non-metric multidimensional scaling (NMDS) and a dissimilarity measure (Bray-Curtis). Centroids are indicated in black boxes. Standard deviation is shown by red (C) or black (G) ellipses. Stress-value = 0.079.
(TIFF)

S7 Fig Relative abundance of bacterial phyla in the rhizosphere of *Avena sativa* L. The values indicated for each treatment are the mean of three replicates (n = 3). C.4D: Mowing/4 days; G.4D: Glyphosate/4 days; C.26D: Mowing/26 days; G.26D: Glyphosate/26 days.
(PDF)

S8 Fig Comparative analysis of mean relative abundance (phylum level) between sampling times. Panel a: Mowing. Panel b: glyphosate suppression. White’s non-parametric t-test was used for statistical analysis (α = 0.05). Category “*Unclassified*” refers to bacterial sequences without taxonomic affiliation. Confidence intervals and *P*-values are indicated in each case.
(TIFF)

S9 Fig Comparative analysis of mean relative abundance (genus level) between suppression methods. White’s non-parametric t-test was used for statistical analysis (α = 0.05). Only those categories with a minimum relative abundance of 0.25% in each sample are shown. Unclassified sequences are not shown for better visualization of profiles. Confidence intervals and *P-*values are indicated. Panel a: without effect size filters (4 days); Panel b: with effect size filter RP > 1.5 (4 days); Panel c: without effect size filters (26 days); Panel d: with effect size filter RP > 1.5 (26 days).
(TIFF)

S10 Fig Heatmaps of OTUs in *Betaproteobacteria* at 4 days (Panel a) and 26 days (Panel b). Cluster analysis was conducted with OTUs with a minimum relative abundance of 0.1% in each sample using UPGMA method.
(TIFF)

S11 Fig Integrative putative model proposed to explain the differences observed between rhizospheric microbial communities from *Avena sativa* plants managed under different suppression methods. The results observed in the rhizosphere of glyphosate-treated plants in our study are summarized in grey boxes. Results shown in white boxes were observed in other plant species from *Poaceae* family (*) or *Fabaceae* family (**) after glyphosate treatments according to literature. AOA: ammonia-oxidizing archaea; AOB: ammonia-oxidizing bacteria; *M*. *loti*: *Mesorhizobium loti*.
(PDF)

S12 Fig Uncropped southern blot images for IncP-1β (4 and 10 days). (TIF)

S13 Fig Uncropped southern blot images for IncP-1β (17 and 26 days). (TIF)

S14 Fig Uncropped southern blot images for IncP-1ε (4 and 10 days). (TIF)

S15 Fig Uncropped southern blot images for IncP-1ε (17 and 26 days). (TIF)

S1 Table Primers used for metabarcoding, qPCR and PCR-Southern blot. (PDF)

S2 Table Contribution of variables to principal component 1 (Dim1) and principal component 2 (Dim2). (PDF)

S3 Table Comparison of multivariate catabolic profiles between suppression methods at each sampling time. The result of ANOSIM test with 999 permutations is indicated.
(PDF)

S4 Table Two-way ANOVA of catabolic diversity indices. *P-*values are indicated for the two factors, suppression method (M) and sampling time (S), and for the interaction (M×S). *df*: degrees of freedom.
(PDF)

S5 Table Number of reads obtained in 454 metabarcoding. (PDF)

S6 Table Two-way ANOVA of alpha-diversity metrics. *P-*values are indicated for main effects (M: suppression method; S: sampling time) and for the interaction (M×S). A *P* < 0.05 indicates statistical significance. *df*: degrees of freedom. Reported *P-*values for M and S correspond to the model without interaction. Chao-1: Chao-1 index (estimated richness); S’: observed richness (number of OTUs); 1/D: Simpson reciprocal index; *H’*: Shannon index; J’: Pielou’s index; *R*_1:2_: Hill-ratio.
(PDF)

S7 Table Goodness of fit statistics of the studied factors fitted to the non-metric multidimensional scaling (NMDS) ordination space of bacterial communities. Values are indicated for each factor. Number of free permutations: 999.
(PDF)

S8 Table Taxonomic affiliation of operative taxonomic units (OTUs) obtained in metabarcoding analysis. Categories with a minimum relative abundance of 0.2% and with a mean ratio of proportions higher than 2 (RP > 2) are shown.
(PDF)

S9 Table Equations of qPCR standard curves. The results for ammonia-oxidizing bacteria (AOB), ammonia-oxidizing archaea (AOA), *Actinobacteria*, total bacteria and Archaea are indicated.
(PDF)

S10 Table Two-way ANOVA of copy numbers for different indicator genes. *P-*values are indicated for each factor (S: sampling time; M: suppression method) and for the interaction (M×S). *df*: degrees of freedom. Only those cases in which no interaction was observed are shown. Reported *P-*values for M and S correspond to the model without interaction.
(PDF)

S11 Table Results of qPCR of *korB* gene in total community DNA. The parameter threshold-cycle (*Ct*) of the amplification curves are indicated for each sample. Standard curve: *Ct* = 48.33–3.16 log_10_ (*korB* copies) (R^2^ = 0.999; Efficiency = 107.14%). NA indicates no amplification.
(PDF)
