# Supplementary material for: Suppression treatment differentially influences the microbial community and the occurrence of broad host range plasmids in the rhizosphere of the model cover crop Avena sativa L
Source: PLoS One. 2019 Oct 9;14(10):e0223600. doi: 10.1371/journal.pone.0223600 (PMC6785065; doi:10.1371/journal.pone.0223600)
Supplement: S1 Text — (PDF) [file pone.0223600.s001.pdf]

## Quantitative Real Time PCR of *Actinobacteria*

The composition of the master mix was as follows: 7.5 µL of PCR iTaq Universal SYBR Green Supermix (2×; Bio-Rad Laboratories); 0.3 µL of each primer (stocks 10 µM, Invitrogen), 1 µL of DNA (~1–10 ng $\mu L^{-1}$) and ultrapure water to 15 µL. The amplification program was as follows: pre-incubation (95°C, 5 min, 1 cycle), amplification (95°C 15 s, 59°C 30 s, 72°C 45 s, 35 cycles), followed by melting curve analysis (65–95 °C). Decimal dilutions of a plasmid harboring one copy of 16S rRNA gene of DNA of *Streptomyces albus* DSM 40313 were used as standards (serial 1/10 dilutions to obtain between $4.97\times10^{6}$ and $4.97\times10^{3}$ copies).

## Quantitative Real Time PCR of Archaea

The composition of the master mix and concentration of reagents was as described above for *Actinobacteria*. The amplification program was as follows: pre-incubation (95°C, 5 min, 1 cycle), amplification (95°C 15 s, 58°C 30 s, 72°C 45 s, 40 cycles), followed by melting curve analysis (65–95 °C). Standard curve was constructed with serial decimal dilutions of the standard (cloned amplification product from DNA sample of an anaerobic digester) to obtain between $7.07\times10^{3}$ and $7.07\times10^{6}$ copies.

## PCR of *trfA* gene for PCR-Southern blot detection of IncP-1 plasmids

The final concentrations of reagents were as follows: True Start buffer 1× (Thermo Scientific), dNTPs 0.2 mM, primers 0.6 µM, $MgCl_2$ 2.5 mM, True Start Taq 0.625 U per reaction (Thermo Scientific) and milliQ water up to 25 µL. One microliter of the purified DNA extracted from rhizospheric soil was used as template. The following amplification program was used: initial step of 5 min at 94°C, 30 cycles of 30 s at 94°C, 20 s at 60°C, 20 s a 72°C and a final elongation step of 5 min a 72°C.
