# Supplementary material for: Suppression treatment differentially influences the microbial community and the occurrence of broad host range plasmids in the rhizosphere of the model cover crop Avena sativa L
Source: PLoS One. 2019 Oct 9;14(10):e0223600. doi: 10.1371/journal.pone.0223600 (PMC6785065; doi:10.1371/journal.pone.0223600)
Supplement: S2 Text — (PDF) [file pone.0223600.s002.pdf]

Hybridization and detection of IncP-1 plasmids

Hybridization was conducted with a buffer of medium astringency (75%-100% homology) (SSC 5X, formamide 20% v/v, blocking agent 2% w/v, sodium N-lauroylsarcosine 0.1% w/v and sodium dodecyl sulfate 0.02% w/v). After hybridization, membranes were washed 5 min two times in a solution of low astringency (SSC 2X, sodium dodecyl sulfate 0.1% w/v) and then in a high astringency solution two times during 15 min at 68°C. Membranes were hybridized with chemiluminescent digoxigenin (DIG)-labeled probes following manufacturer instructions (Roche Diagnostic, Mannheim, Germany).

The following solutions were used for the detection of hybridized membranes: DIG1 (100 mM maleic acid; 150 mM NaCl pH 7.5), DIG3 (100 mM Tris-HCl; 100 mM NaCl pH 9.5), blocking reagent (casein, 10% w/v in solution DIG1), antibody (Anti-Digoxigenin-AP Fab fragments, Roche Diagnostics GmbH, Mannheim, Germany, Cat# 11 093 274 910, RRID:AB_2313640, polyclonal), Buffer 1 (DIG1 + 0.3% v/v Tween 20), Buffer 2 (1% w/v blocking agent in DIG1), Buffer 3 (Buffer 2 + antibody at 1:10000 ratio). The protocol for detection was as follows: 1) washing of the membrane with Buffer 1 (50 mL) 5 min at room temperature; 2) Blocking of the membrane 30 min at room temperature using 100 mL of Buffer 2 3) Exposure of blocked membrane to the antibody (20 mL, 30 min at room temperature) 4) Washing of excess of antibody two times (15 min) with 100 mL of Buffer 1 at room temperature 5) Exposure of membrane to Buffer 3 (20 mL, 5 min, two times) 6) Exposure to 900 µL of CDP Star substrate (0.25 mM, ready to use, Roche) 6) Sealing of the membrane 7) Autoradiography.
