# Supplementary figures and images for: Suppression treatment differentially influences the microbial community and the occurrence of broad host range plasmids in the rhizosphere of the model cover crop Avena sativa L
Source: PLoS One. 2019 Oct 9;14(10):e0223600. doi: 10.1371/journal.pone.0223600 (PMC6785065; doi:10.1371/journal.pone.0223600)

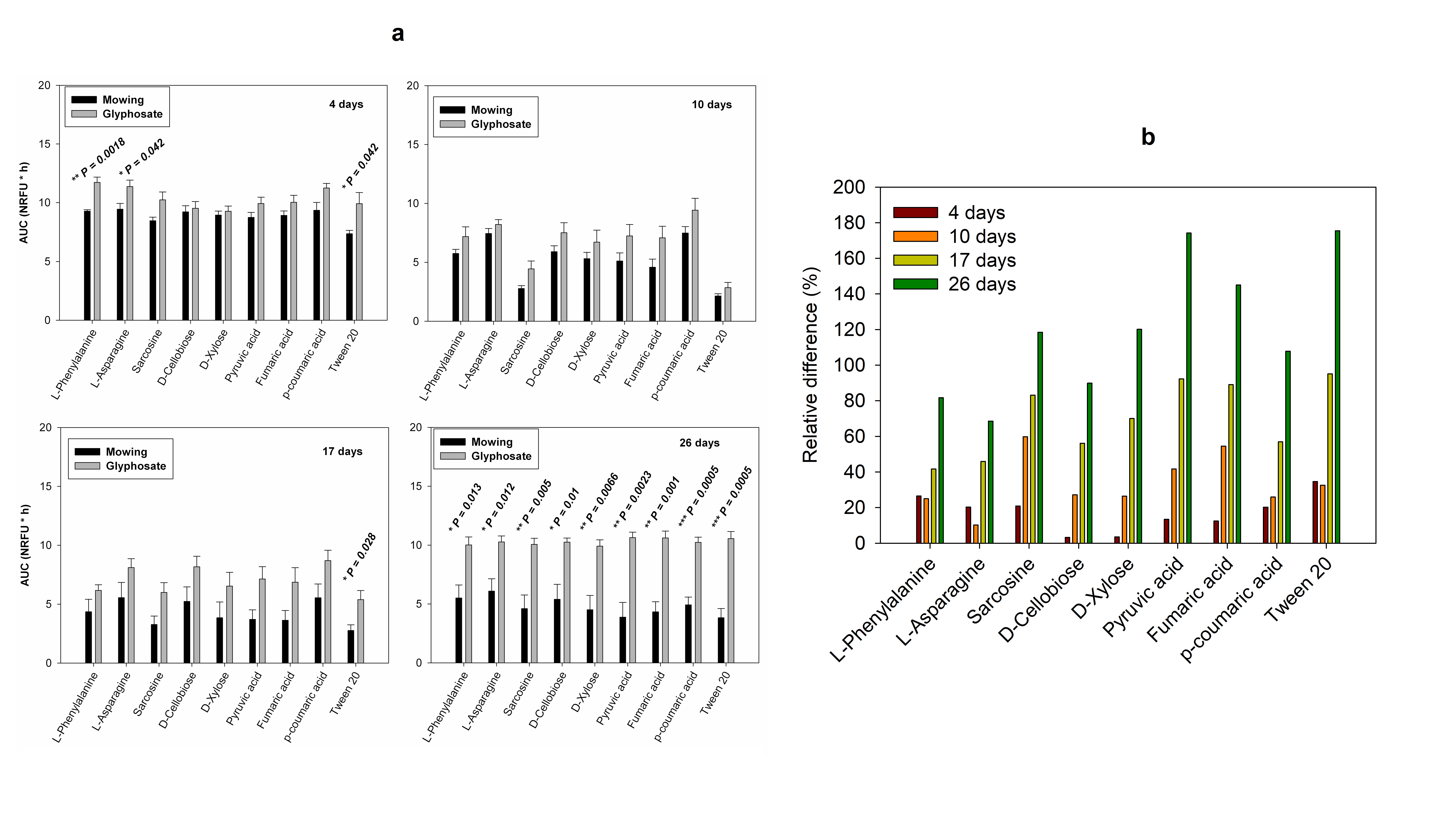

Supplement: S1 Fig — Panel a = Absolute responses; Panel b: Relative differences. Error bars indicate the standard error of the mean (n = 4). The P-value is shown only for statistically significant differences (asterisk, two-sample t-test, P < 0.05). AUC: integrated area under the curve. NRFU: normalized relative fluorescence units. (TIFF) [file pone.0223600.s004.tiff]

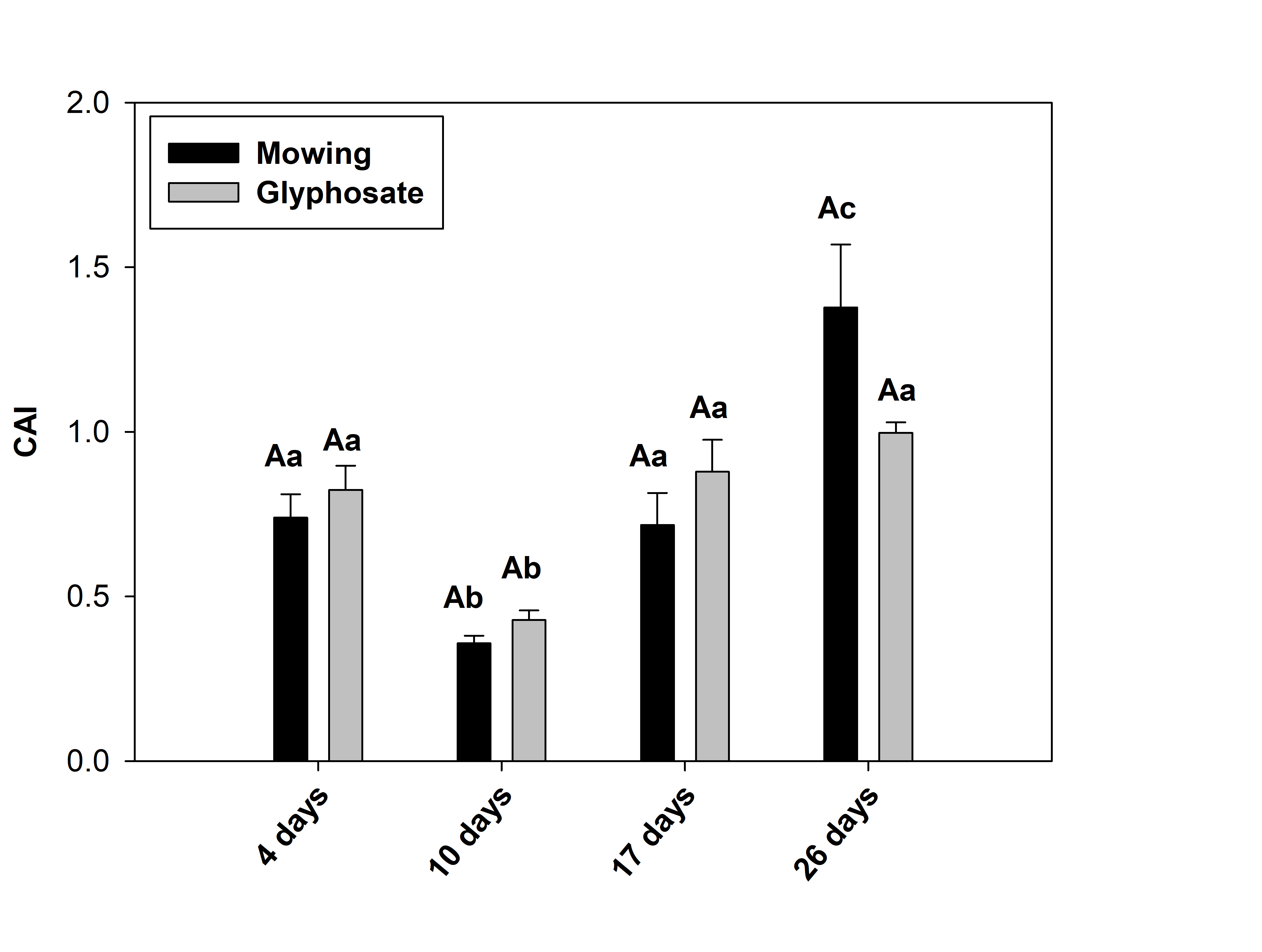

Supplement: S2 Fig — Upper case letters indicate statistically significant differences between suppression methods at each sampling time. Lower case letters indicate statistically significant differences among sampling times within a suppression method (P < 0.05, Tukey’s HSD test). (TIFF) [file pone.0223600.s005.TIFF]

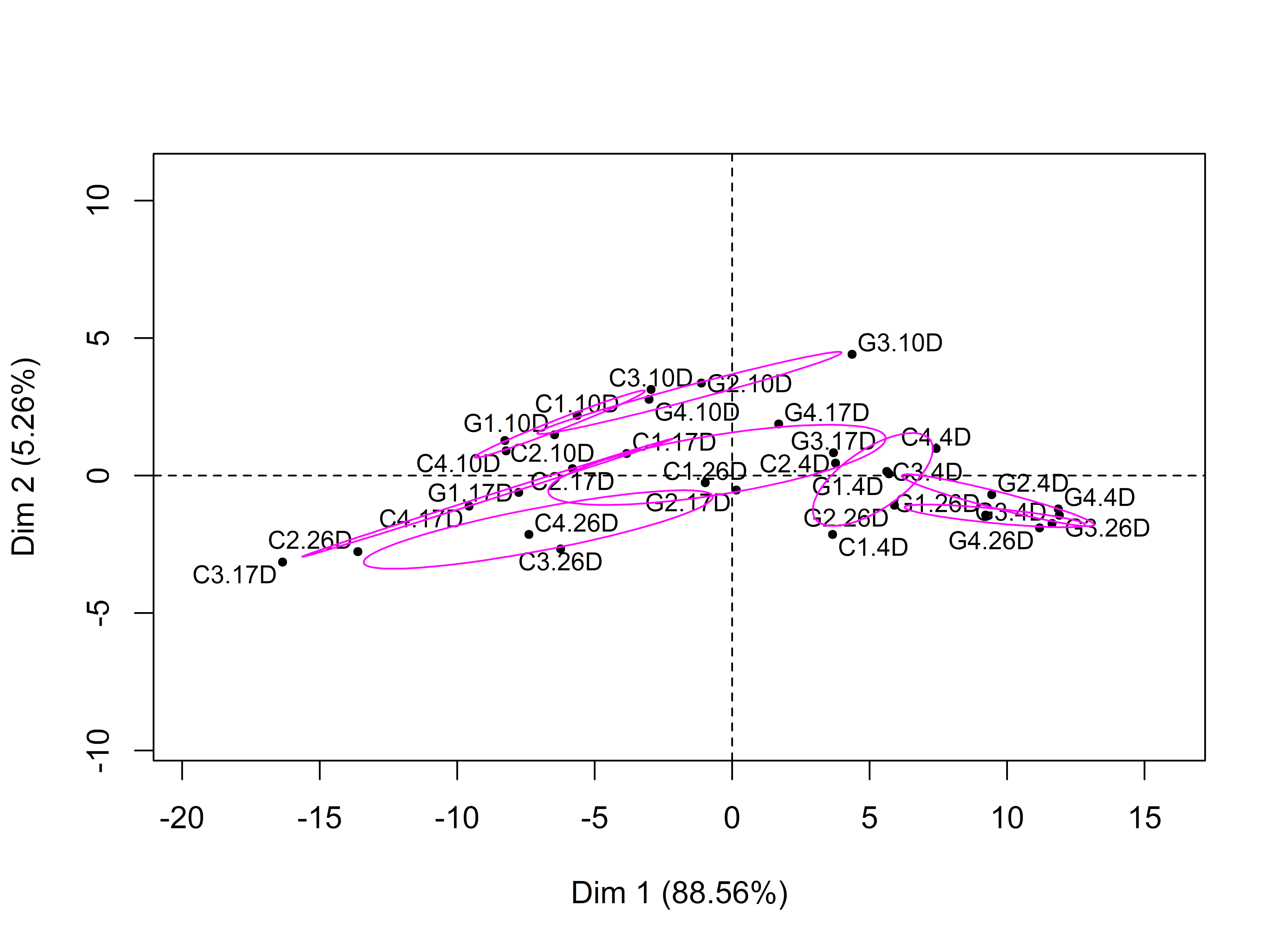

Supplement: S3 Fig — Confidence ellipses (95%) around replicates with a same treatment are indicated in purple. Treatments: 1–4: Mowing/4 days; 5–8: Glyphosate/4 days; 9–12: Mowing/10 days; 13–16: Glyphosate/10 days; 17–20: Mowing/17 days; 21–24: Glyphosate/17 days; 25–28: Mowing/26 days; 29–32: Glyphosate/26 days. (TIFF) [file pone.0223600.s006.tiff]

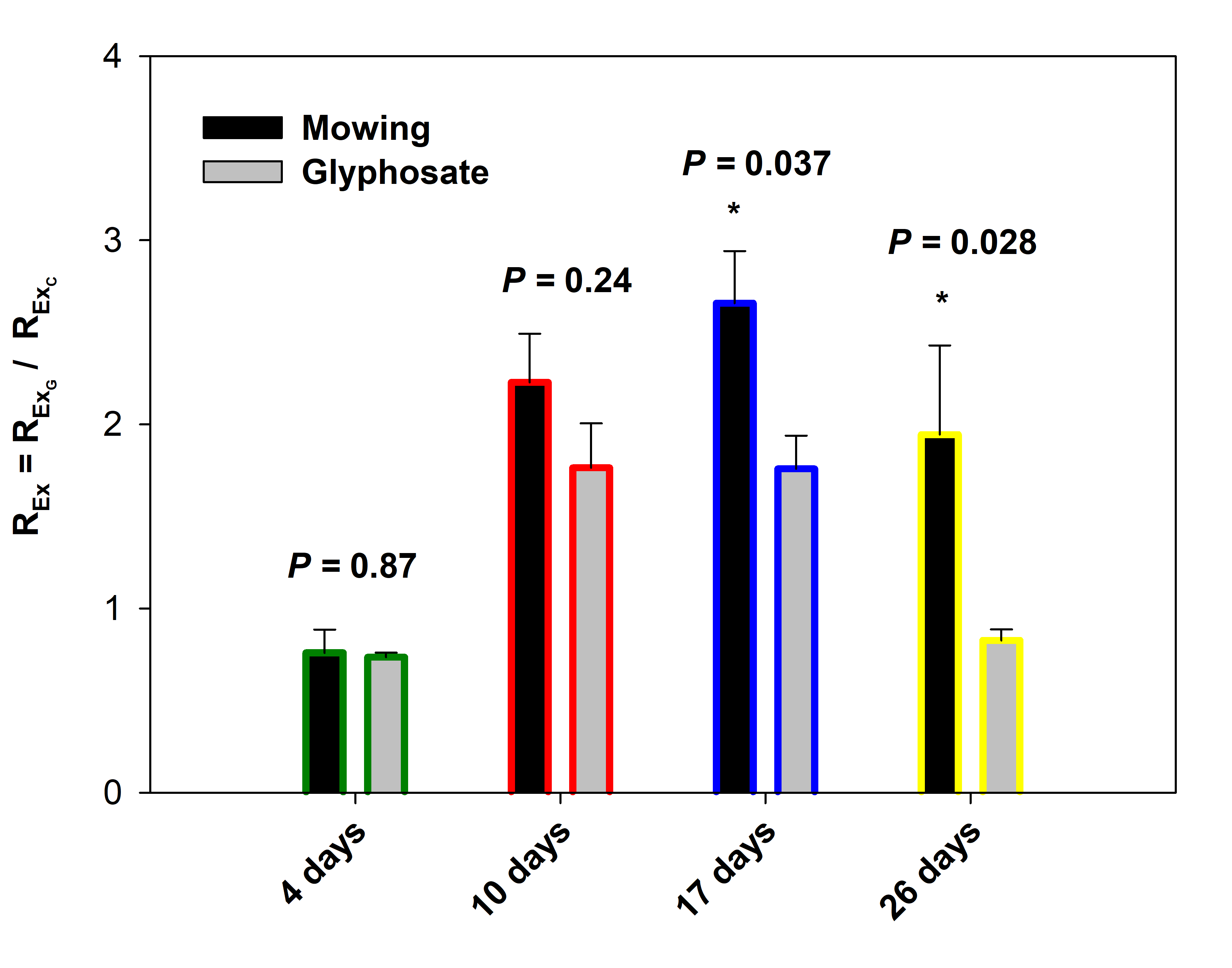

Supplement: S4 Fig — The ratio of responses to root exudates (REx = Response to ExG / Response to ExC) is shown for microbial communities of cut plants and glyphosate-treated plants. Error bars indicate the standard error of the mean (n = 4). Asterisks indicate significant differences according to two-sample t-test (P < 0.05), except for data at 26 days (Wilcoxon test, P < 0.05). Colours indicate that root exudates tested at 4, 10, 17 and 26 days were collected at the corresponding times, thus, no comparisons of responses were made among sampling times. (TIFF) [file pone.0223600.s007.tiff]

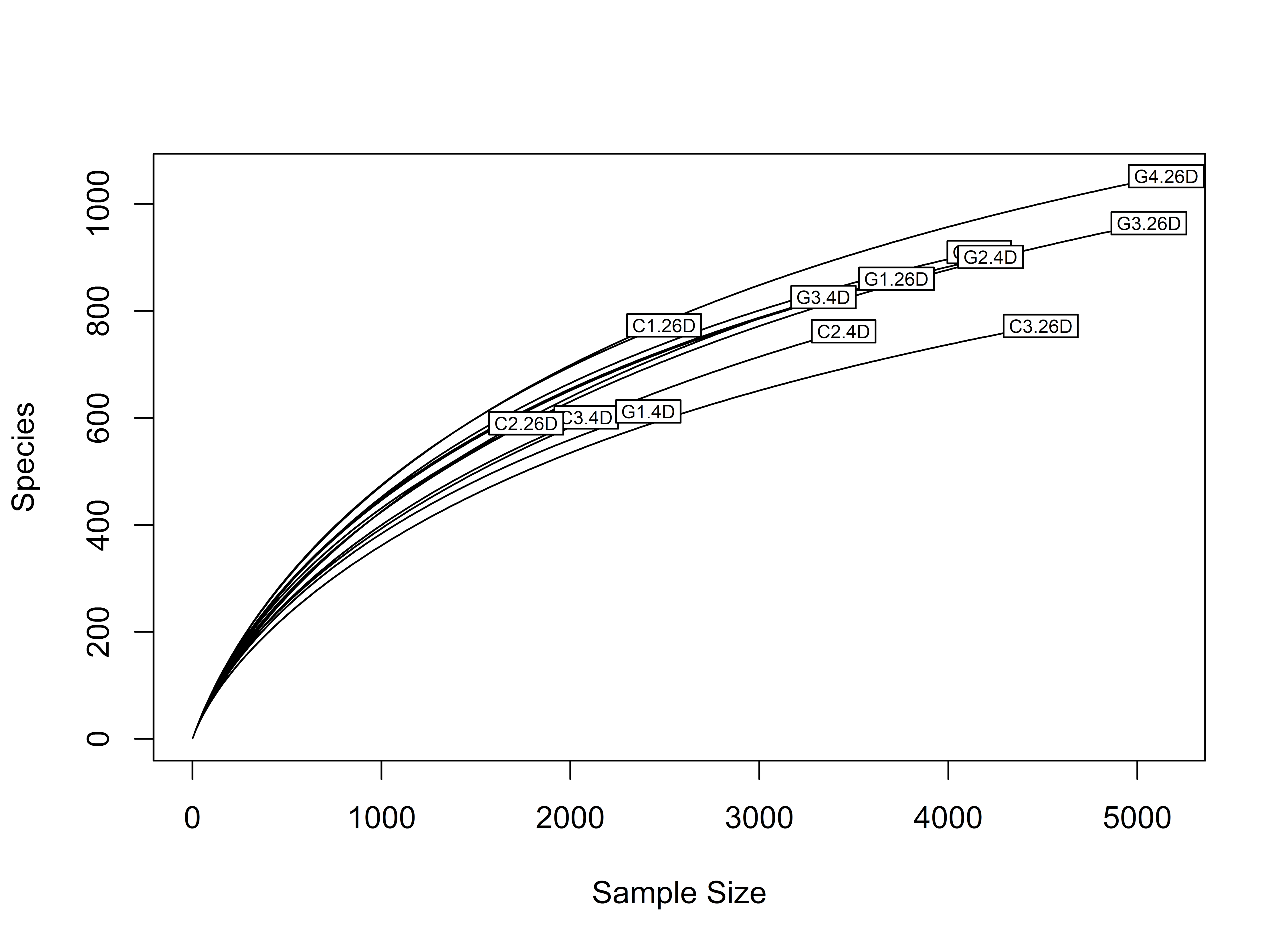

Supplement: S5 Fig — The number of operative taxonomic units (OTUs) of Bacteria is indicated for an increasing sampling effort (“sample size”). (TIFF) [file pone.0223600.s008.tiff]

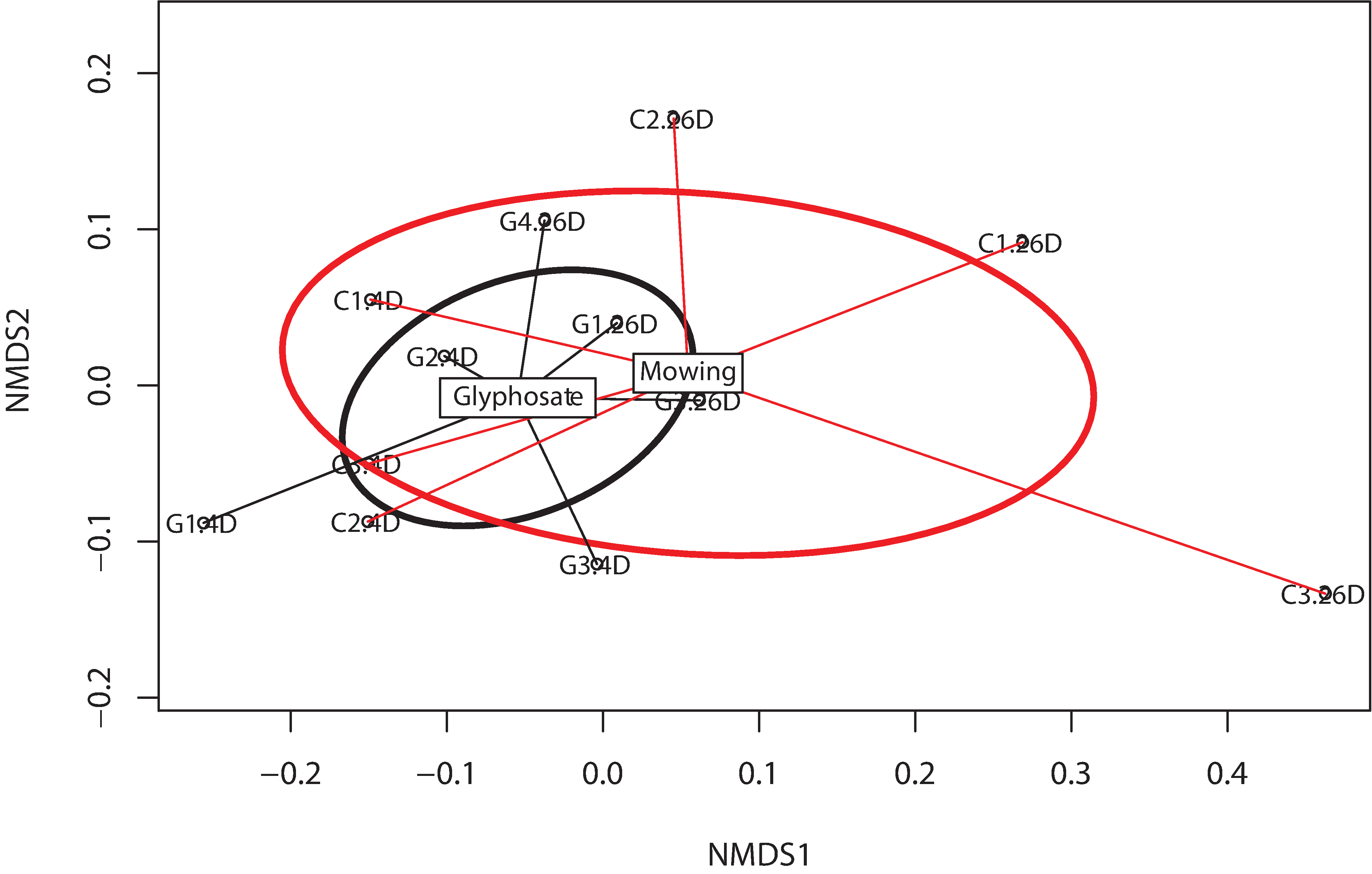

Supplement: S6 Fig — Centroids are indicated in black boxes. Standard deviation is shown by red (C) or black (G) ellipses. Stress-value = 0.079. (TIFF) [file pone.0223600.s009.tiff]

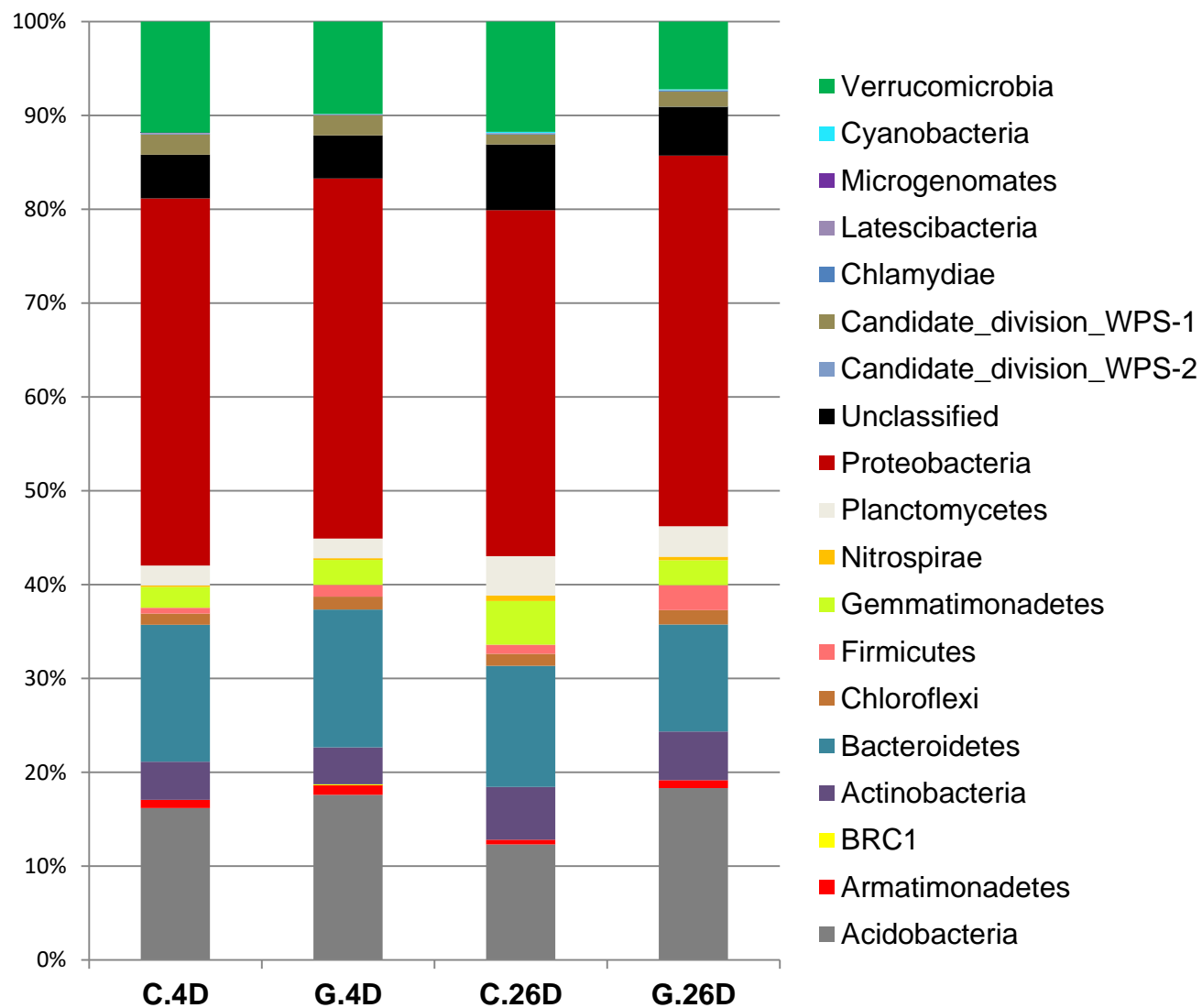

Supplement: S7 Fig — The values indicated for each treatment are the mean of three replicates (n = 3). C.4D: Mowing/4 days; G.4D: Glyphosate/4 days; C.26D: Mowing/26 days; G.26D: Glyphosate/26 days. (PDF) [file pone.0223600.s010.pdf]

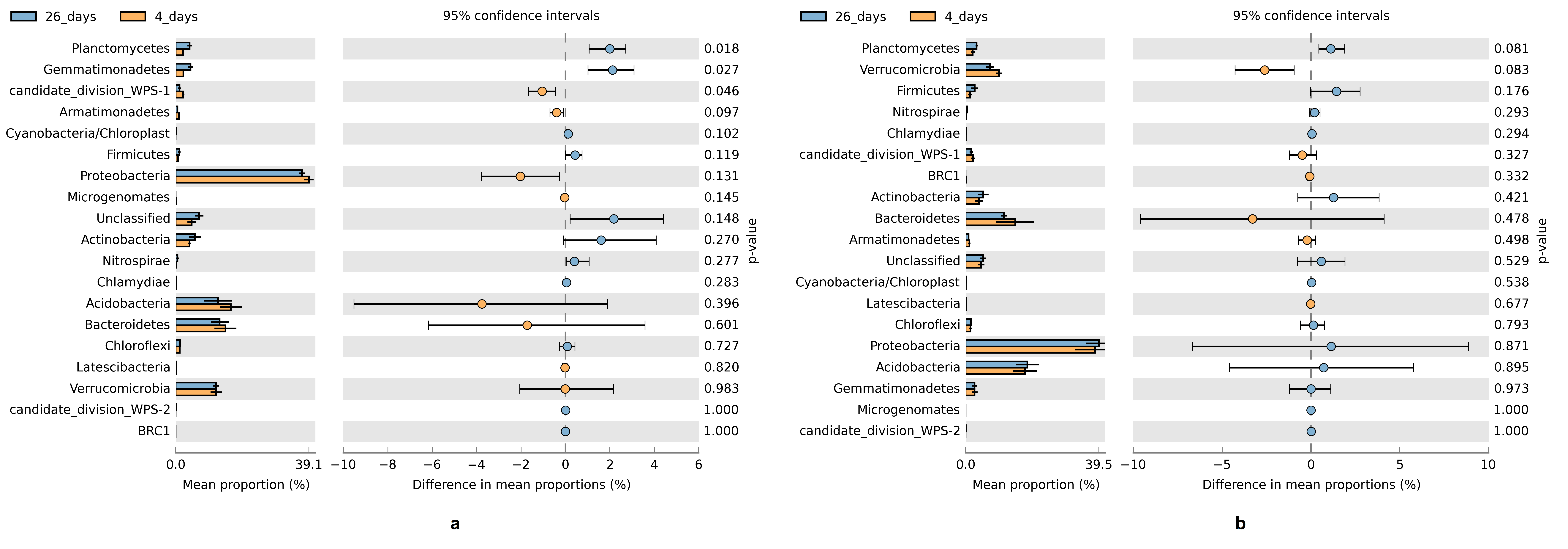

Supplement: S8 Fig — Panel a: Mowing. Panel b: glyphosate suppression. White’s non-parametric t-test was used for statistical analysis (α = 0.05). Category “Unclassified” refers to bacterial sequences without taxonomic affiliation. Confidence intervals and P-values are indicated in each case. (TIFF) [file pone.0223600.s011.tiff]

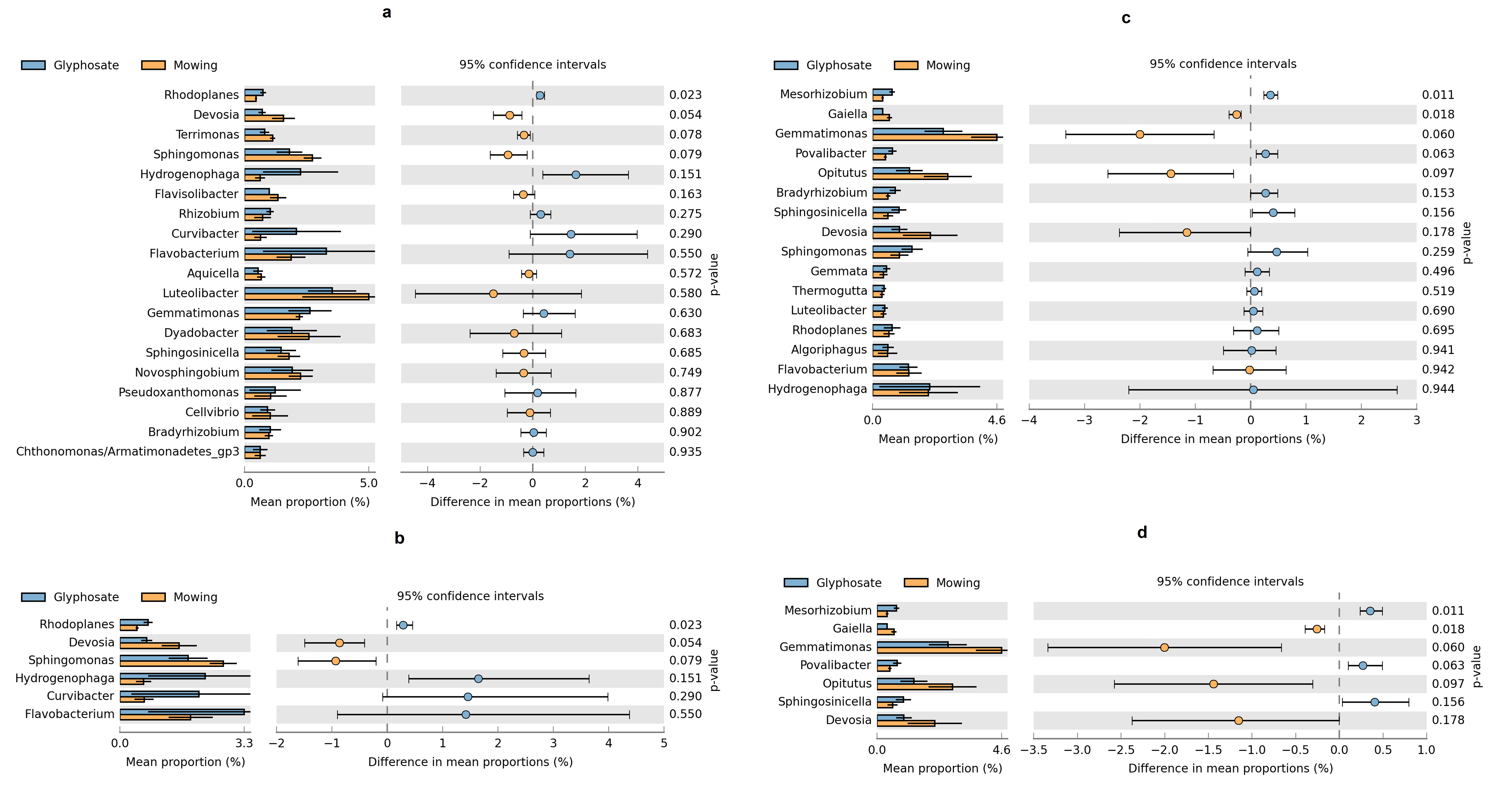

Supplement: S9 Fig — White’s non-parametric t-test was used for statistical analysis (α = 0.05). Only those categories with a minimum relative abundance of 0.25% in each sample are shown. Unclassified sequences are not shown for better visualization of profiles. Confidence intervals and P-values are indicated. Panel a: without effect size filters (4 days); Panel b: with effect size filter RP > 1.5 (4 days); Panel c: without effect size filters (26 days); Panel d: with effect size filter RP > 1.5 (26 days). (TIFF) [file pone.0223600.s012.tiff]

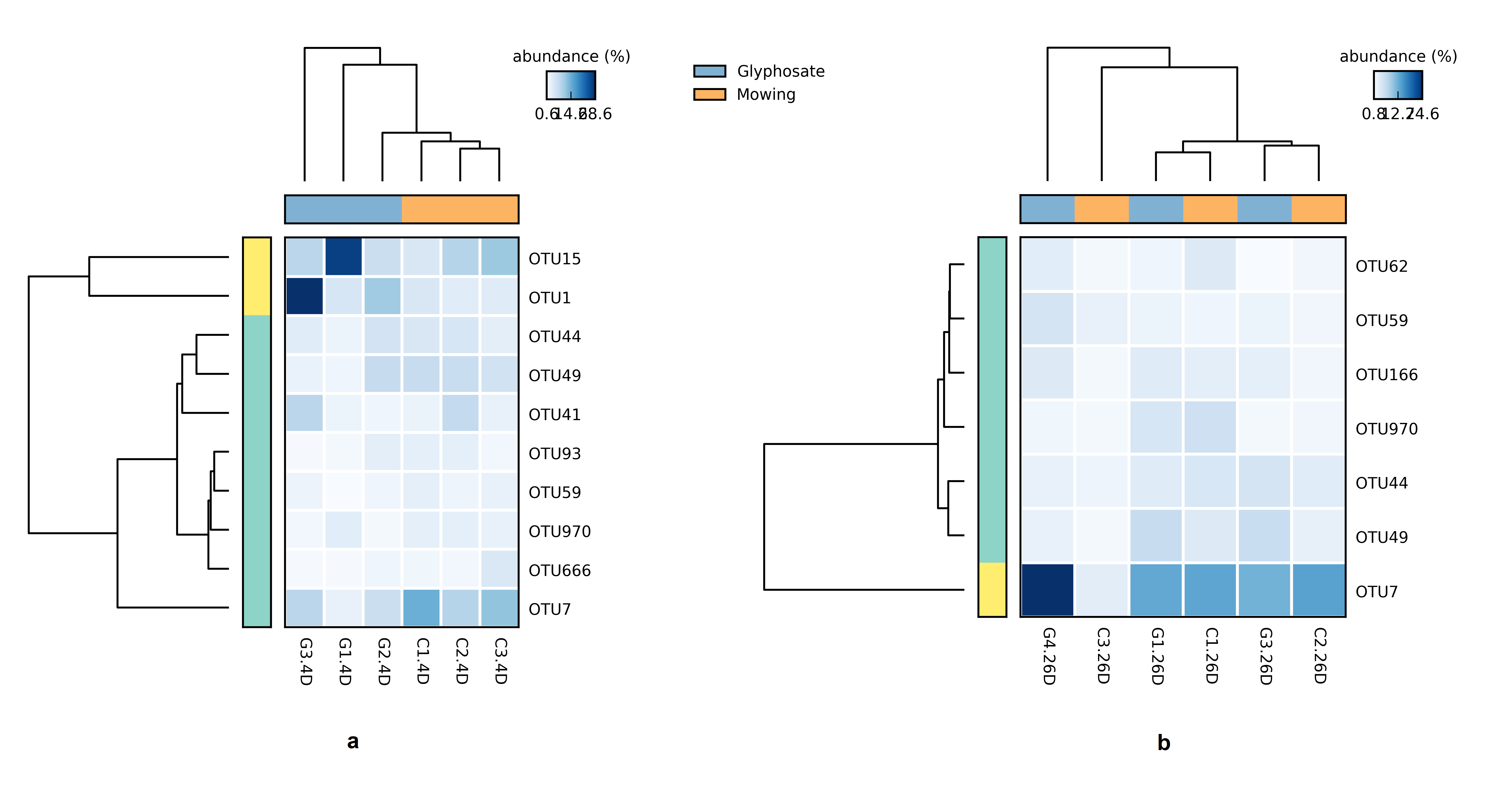

Supplement: S10 Fig — Cluster analysis was conducted with OTUs with a minimum relative abundance of 0.1% in each sample using UPGMA method. (TIFF) [file pone.0223600.s013.tiff]

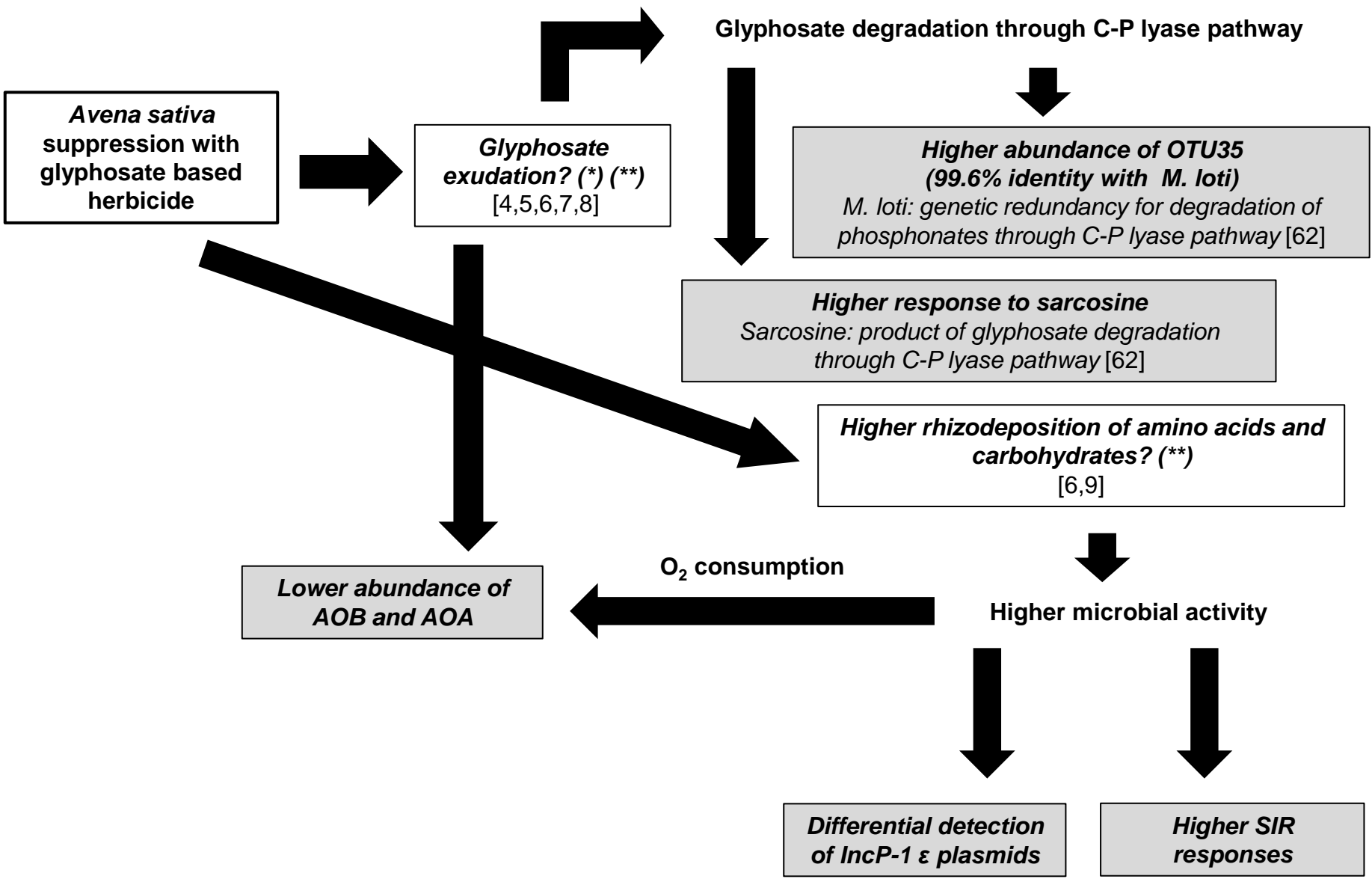

Supplement: S11 Fig — The results observed in the rhizosphere of glyphosate-treated plants in our study are summarized in grey boxes. Results shown in white boxes were observed in other plant species from Poaceae family (*) or Fabaceae family (**) after glyphosate treatments according to literature. AOA: ammonia-oxidizing archaea; AOB: ammonia-oxidizing bacteria; M. loti: Mesorhizobium loti. (PDF) [file pone.0223600.s014.pdf]

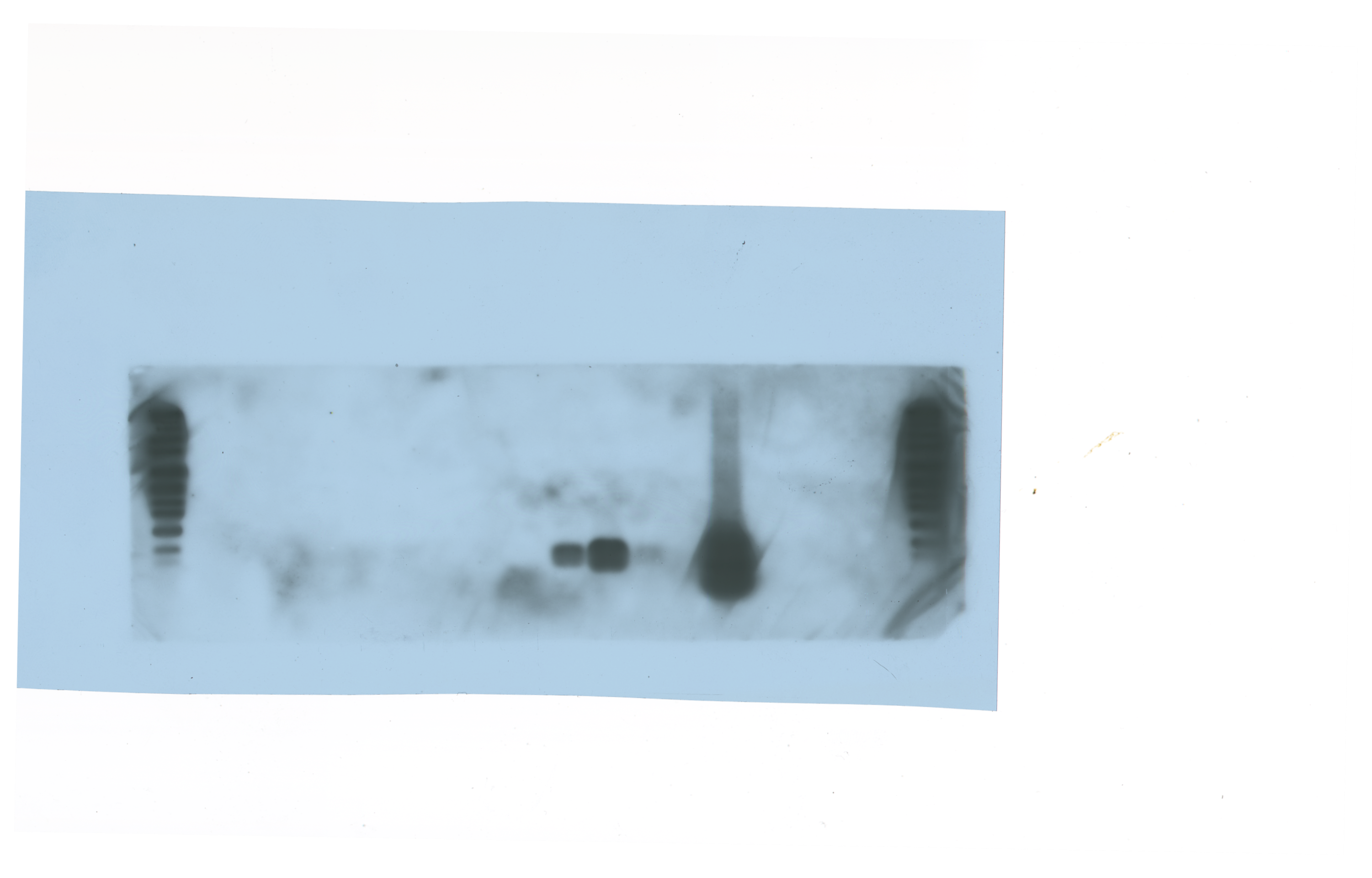

Supplement: S12 Fig — (TIF) [file pone.0223600.s015.tif]

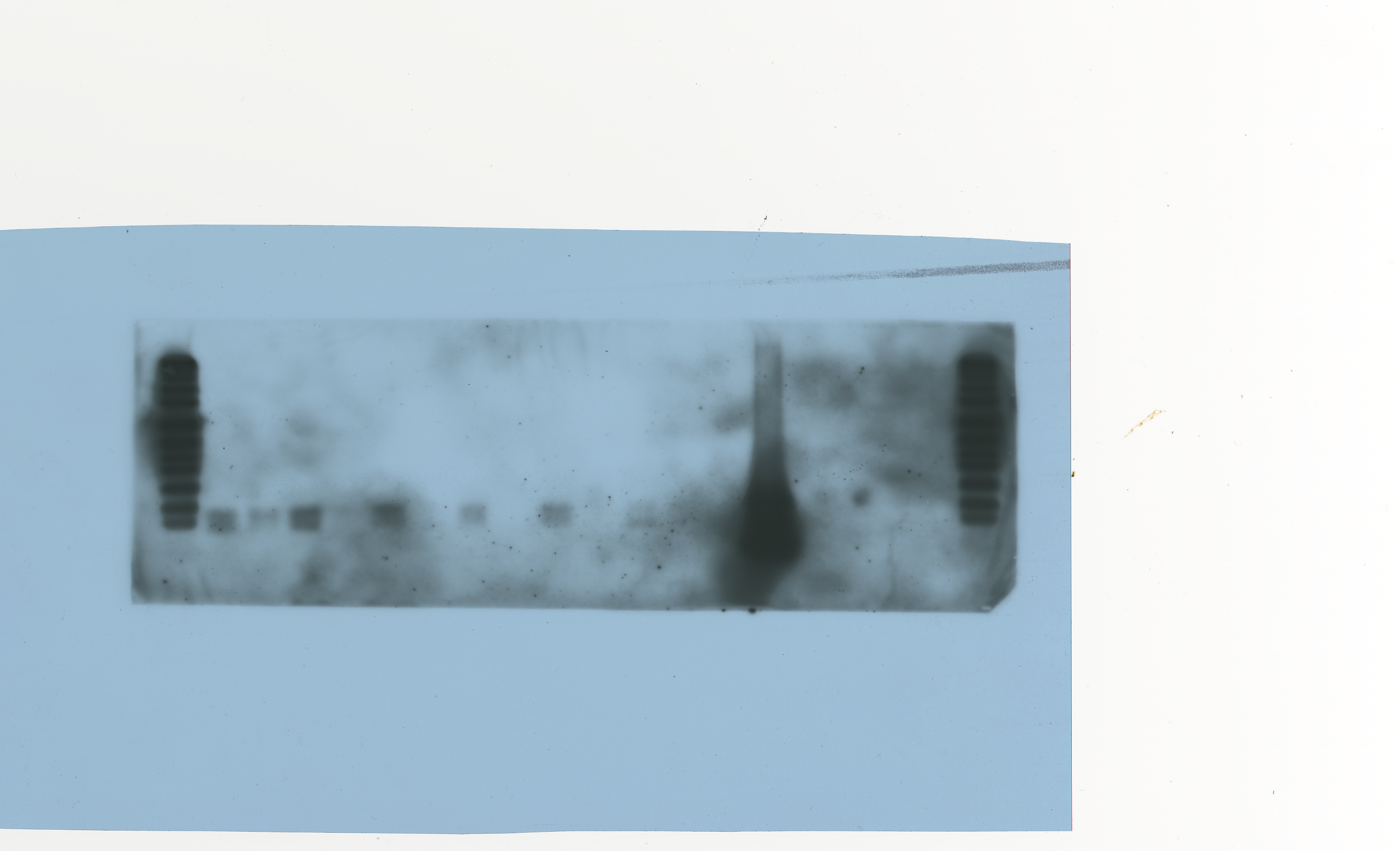

Supplement: S13 Fig — (TIF) [file pone.0223600.s016.tif]

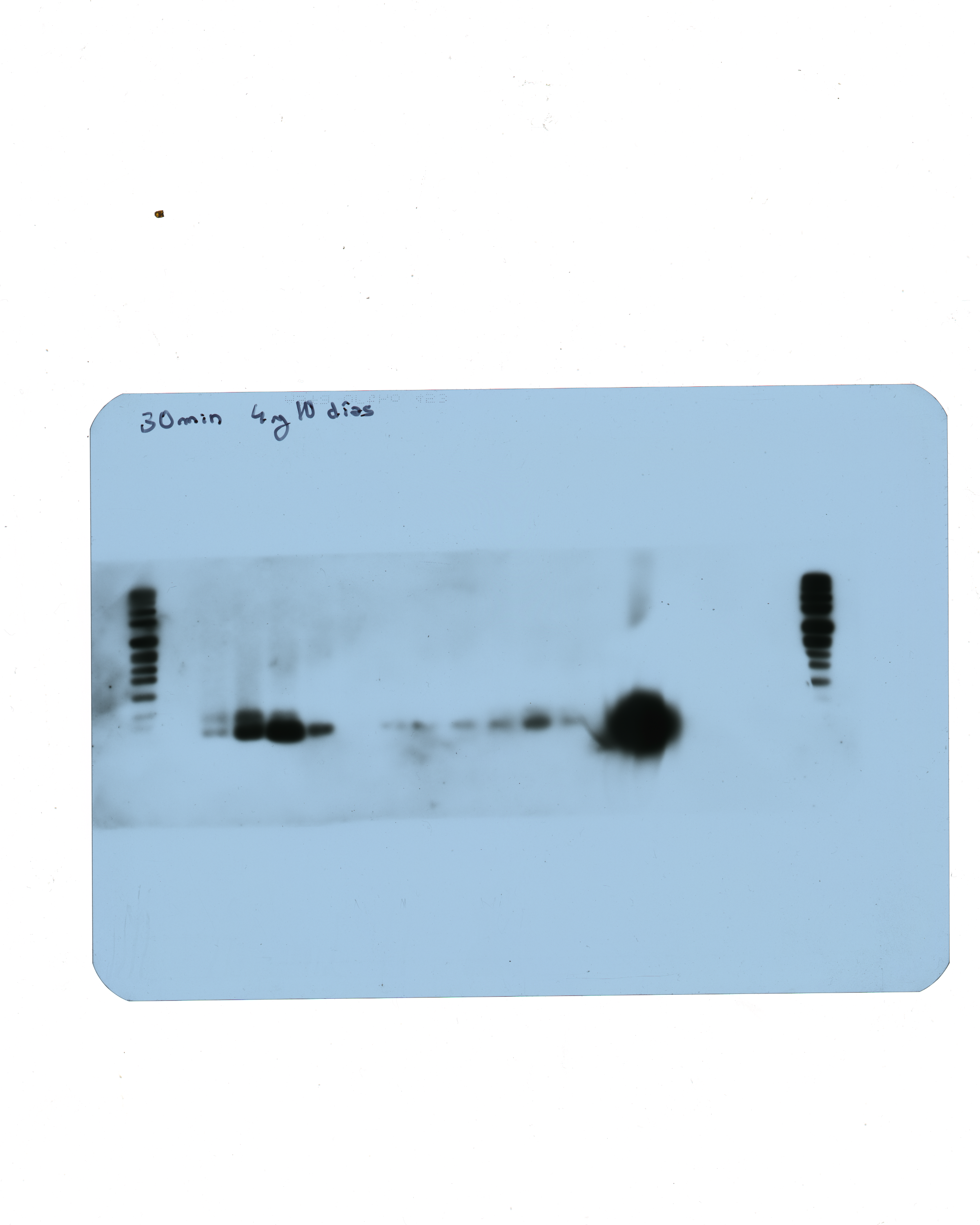

Supplement: S14 Fig — (TIF) [file pone.0223600.s017.tif]

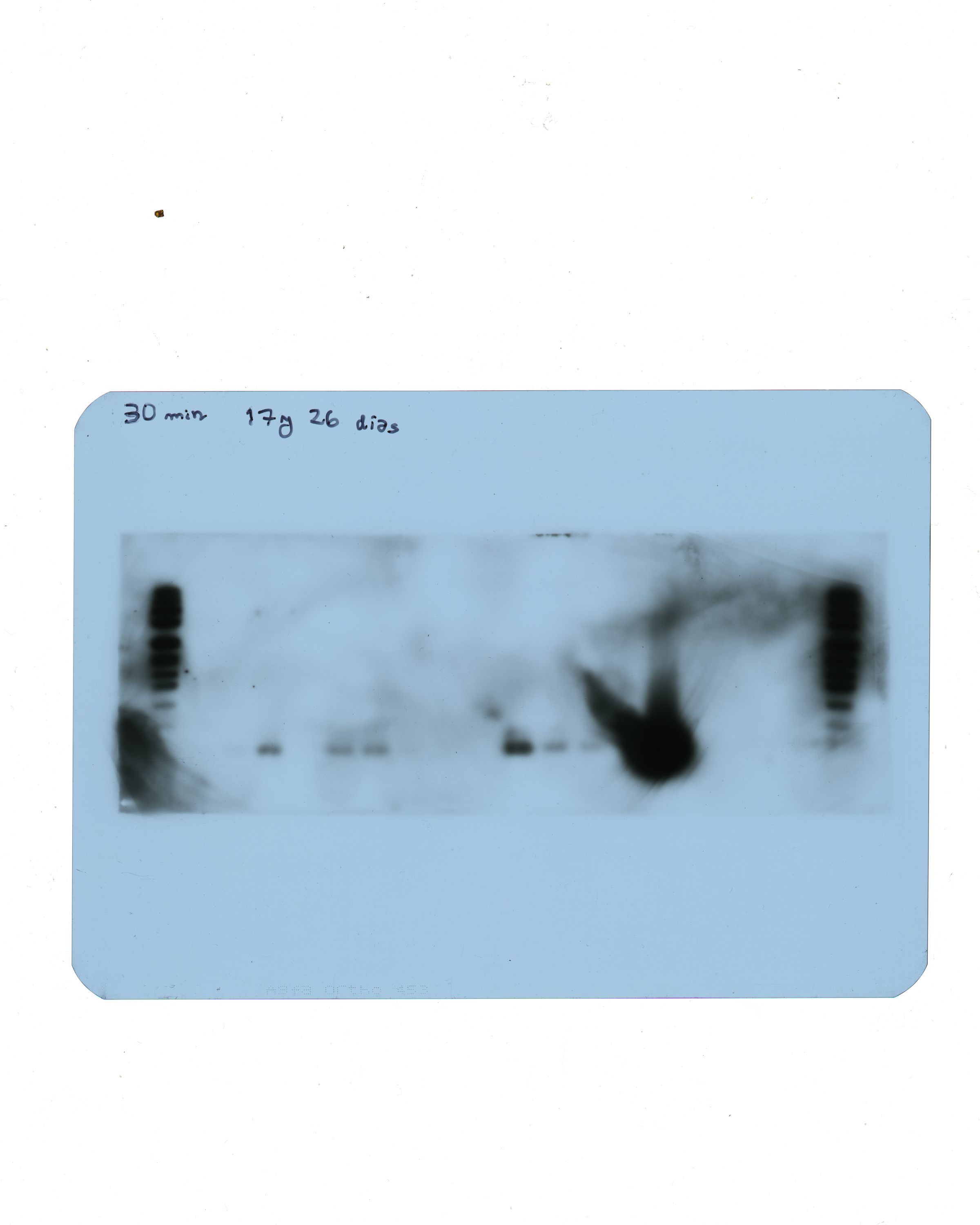

Supplement: S15 Fig — (TIF) [file pone.0223600.s018.tif]
