# Supplementary material for: Suppression treatment differentially influences the microbial community and the occurrence of broad host range plasmids in the rhizosphere of the model cover crop Avena sativa L
Source: PLoS One. 2019 Oct 9;14(10):e0223600. doi: 10.1371/journal.pone.0223600 (PMC6785065; doi:10.1371/journal.pone.0223600)
Supplement: S1 Table — (PDF) [file pone.0223600.s019.pdf]

Primers	Sequence (5′ – 3′)	Gene	Reference	Group
**515F**	GTGCCAGCMGCCGCGGTAA	16S rRNA	[32]	Total bacteria
**806R**	GGACTACVSGGGTATCTAAT			
**338F**	ACTCCTACGGGAGGCAGCAG	16S rRNA	[43]	Total bacteria
**518R**	ATTACCGCGGCTGCTGG			
**amoA-1F**	GGGGTTTCTACTGGTGGT	*amoA*$_{AOB}$	[42]	AOB
**amoA-2R**	CCCCTCKGSAAAGCCTTCTTC			
**amoA-19F**	ATGGTCTGGCTWAGACG	*amoA*$_{AOA}$	[40]	AOA
**CrenamoA616r48x**	GCCATCCABCKRTANGTCCA		[41]	
**P-Acti-1154-a-S-19**	GRDACYGCCGGGGTYAACT	16S rRNA	[44]	*Actinobacteria*
**S-P-Acti-1339-a-A-18**	TCWGCGATTACTAGCGAC			
**F**	TCATCGACAACGACTACAACG	*korB*	[48]	IncP-1 plasmids (all subgroups)
**R**	TTCTTCTTGCCCTTCGCCAG			
**Fz**	TCGTGGATAACGACTACAACG			
**Rge**	TTYTTCYTGCCCTTGGCCAG			
**Rd**	TTCTTGACTCCCTTCGCCAG			
**trfA733f**	TTCACSTTCTACGAGMTKTGCCAGGAC	*trfA*	[49]	IncP-1 plasmids *(*α, β, ε*)*
**trfA1013r**	GWCAGCTTGCGGTACTTCTCCCA			
**Arch346aF**	CGGGGYGCASCAGGCGCGA	16S rRNA	[45]	Archaea
**Arch934b**	GTGCTCCCCCGCCAATTCCT			
