# Supplementary material for: Suppression treatment differentially influences the microbial community and the occurrence of broad host range plasmids in the rhizosphere of the model cover crop Avena sativa L
Source: PLoS One. 2019 Oct 9;14(10):e0223600. doi: 10.1371/journal.pone.0223600 (PMC6785065; doi:10.1371/journal.pone.0223600)
Supplement: S2 Table — (PDF) [file pone.0223600.s020.pdf]

**Variables**	**Contribution (%)**	
	**Dim.1**	**Dim.2**
L-Phenylalanine	11.21	1.17×10^{-6}
L-Asparagine	7.69	4.14
Sarcosine	14.09	14.84
D-Cellobiose	8.11	4.69
D-Xylose	10.22	5.26
Pyruvic acid	12.88	4.04
Fumaric acid	13.03	0.271
p-coumaric acid	7.92	19.95
Tween 20	14.83	46.81
