# Supplementary material for: Suppression treatment differentially influences the microbial community and the occurrence of broad host range plasmids in the rhizosphere of the model cover crop Avena sativa L
Source: PLoS One. 2019 Oct 9;14(10):e0223600. doi: 10.1371/journal.pone.0223600 (PMC6785065; doi:10.1371/journal.pone.0223600)
Supplement: S3 Table — The result of ANOSIM test with 999 permutations is indicated. (PDF) [file pone.0223600.s021.pdf]

	ANOSIM (Mowing *vs* Glyphosate)			
	4 days	10 days	17 days	26 days
**Significance**	0.062	0.17	0.15	0.029
**R-Statistic**	0.44	0.10	0.23	0.68
