# Supplementary material for: Suppression treatment differentially influences the microbial community and the occurrence of broad host range plasmids in the rhizosphere of the model cover crop Avena sativa L
Source: PLoS One. 2019 Oct 9;14(10):e0223600. doi: 10.1371/journal.pone.0223600 (PMC6785065; doi:10.1371/journal.pone.0223600)
Supplement: S4 Table — P-values are indicated for the two factors, suppression method (M) and sampling time (S), and for the interaction (M×S). df: degrees of freedom. (PDF) [file pone.0223600.s022.pdf]

**Factor**	*Evenness (E)*	*Shannon (H')*
**M (*df = 1*)**	$P = 0.0001$	$P = 0.0003$
**S (*df = 3*)**	$P < 0.0001$	$P < 0.0001$
**M×S (*df = 3*)**	$P = 0.027$	$P = 0.052$
