# Supplementary material for: Suppression treatment differentially influences the microbial community and the occurrence of broad host range plasmids in the rhizosphere of the model cover crop Avena sativa L
Source: PLoS One. 2019 Oct 9;14(10):e0223600. doi: 10.1371/journal.pone.0223600 (PMC6785065; doi:10.1371/journal.pone.0223600)
Supplement: S5 Table — (PDF) [file pone.0223600.s023.pdf]

Sample	Number of reads Bacteria	Number of reads Archaea	Number of reads without affiliation	Total number of reads	% reads Bacteria	% reads Archaea	% reads without affiliation
C1.4D	4480	53	0	4533	98.83	1.17	0
C2.4D	3687	42	0	3729	98.87	1.13	0
C3.4D	2249	19	0	2268	99.16	0.84	0
G1.4D	2530	20	0	2550	99.22	0.78	0
G2.4D	4580	50	1	4631	98.90	1.08	0.02
G3.4D	3577	40	0	3617	98.89	1.11	0
C1.26D	2814	30	0	2844	98.95	1.05	0
C2.26D	1966	24	0	1990	98.79	1.21	0
C3.26D	4802	62	0	4864	98.73	1.27	0
G1.26D	4012	41	0	4053	98.99	1.01	0
G3.26D	5422	63	0	5485	98.85	1.15	0
G4.26D	5605	68	2	5675	98.77	1.20	0.03
