# Supplementary material for: Suppression treatment differentially influences the microbial community and the occurrence of broad host range plasmids in the rhizosphere of the model cover crop Avena sativa L
Source: PLoS One. 2019 Oct 9;14(10):e0223600. doi: 10.1371/journal.pone.0223600 (PMC6785065; doi:10.1371/journal.pone.0223600)
Supplement: S6 Table — P-values are indicated for main effects (M: suppression method; S: sampling time) and for the interaction (M×S). A P < 0.05 indicates statistical significance. df: degrees of freedom. Reported P-values for M and S correspond to the model without interaction. Chao-1: Chao-1 index (estimated richness); S’: observed richness (number of OTUs); 1/D: Simpson reciprocal index; H’: Shannon index; J’: Pielou’s index; R1:2: Hill-ratio. (PDF) [file pone.0223600.s024.pdf]

Alpha-diversity metrics						
**Factors**	**Chao-1**	**S’**	**1/D**	***H’***	$\boldsymbol{R_{1:2}}$	**J'**
**M (*df* = 1)**	*P = 0.27*	*P = 0.38*	*P = 0.59*	*P = 0.48*	*P = 0.99*	*P = 0.57*
**S (*df* = 1)**	*P = 0.97*	*P = 0.39*	*P = 0.13*	*P = 0.29*	*P = 0.21*	*P = 0.26*
**M×S (*df* = 1)**	*P = 0.60*	*P = 0.43*	*P = 0.65*	*P = 0.45*	*P = 0.62*	*P = 0.50*
