# Supplementary material for: Suppression treatment differentially influences the microbial community and the occurrence of broad host range plasmids in the rhizosphere of the model cover crop Avena sativa L
Source: PLoS One. 2019 Oct 9;14(10):e0223600. doi: 10.1371/journal.pone.0223600 (PMC6785065; doi:10.1371/journal.pone.0223600)
Supplement: S7 Table — Values are indicated for each factor. Number of free permutations: 999. (PDF) [file pone.0223600.s025.pdf]

Goodness of fit (NMDS-Bray-Curtis dissimilarity)		
**Factor**	R^2	***P-value***
Suppression method	0.068	0.51
Sampling time	0.45	0.005

Goodness of fit (NMDS-Generalized UniFrac)		
**Factor**	R^2	***P-value***
Suppression method	0.05	0.61
Sampling time	0.48	0.005
