# Supplementary material for: Suppression treatment differentially influences the microbial community and the occurrence of broad host range plasmids in the rhizosphere of the model cover crop Avena sativa L
Source: PLoS One. 2019 Oct 9;14(10):e0223600. doi: 10.1371/journal.pone.0223600 (PMC6785065; doi:10.1371/journal.pone.0223600)
Supplement: S8 Table — Categories with a minimum relative abundance of 0.2% and with a mean ratio of proportions higher than 2 (RP > 2) are shown. (PDF) [file pone.0223600.s026.pdf]

| OTU | Lower taxonomic level (RDP classifier) | Confidence (%) | Taxonomic affiliation |
|---|---|---|---|
| OTU1 | Genus | 100 | *Hydrogenophaga* sp. |
| OTU14 | Genus | 100 | *Pseudoxanthomonas* sp. |
| OTU15 | Genus | 48 | *Curvibacter* sp. |
| OTU23 | Order | 99 | *Acidobacteria_Gp4* |
| OTU35 | Genus | 91 | *Mesorhizobium* sp. |
| OTU38 | Genus | 84 | *Luteolibacter* sp. |
| OTU45 | Class | 100 | *Acidobacteria_Gp3* |
