# Supplementary material for: Suppression treatment differentially influences the microbial community and the occurrence of broad host range plasmids in the rhizosphere of the model cover crop Avena sativa L
Source: PLoS One. 2019 Oct 9;14(10):e0223600. doi: 10.1371/journal.pone.0223600 (PMC6785065; doi:10.1371/journal.pone.0223600)
Supplement: S9 Table — The results for ammonia-oxidizing bacteria (AOB), ammonia-oxidizing archaea (AOA), Actinobacteria, total bacteria and Archaea are indicated. (PDF) [file pone.0223600.s027.pdf]

Gene	Group	Equation	R^2	Efficiency (%)
amoA	AOB	$Ct = 36.74 – 3.85 \log_{10} (copy\ number)$	0.996	81.97
amoA	AOA	$Ct = 38.63 – 3.94 \log_{10} (copy\ number)$	0.998	79.46
16S rRNA	*Actinobacteria*	$Ct = 38.02 – 3.41 \log_{10} (copy\ number)$	0.998	96.29
16S rRNA	Total bacteria	$Ct = 41.83 – 3.68 \log_{10} (copy\ number)$	0.993	86.96
16S rRNA	Archaea	$Ct = 36.74 – 3.67 \log_{10} (copy\ number)$	0.998	87.27
