# Supplementary material for: Suppression treatment differentially influences the microbial community and the occurrence of broad host range plasmids in the rhizosphere of the model cover crop Avena sativa L
Source: PLoS One. 2019 Oct 9;14(10):e0223600. doi: 10.1371/journal.pone.0223600 (PMC6785065; doi:10.1371/journal.pone.0223600)
Supplement: S10 Table — P-values are indicated for each factor (S: sampling time; M: suppression method) and for the interaction (M×S). df: degrees of freedom. Only those cases in which no interaction was observed are shown. Reported P-values for M and S correspond to the model without interaction. (PDF) [file pone.0223600.s028.pdf]

**Factor**	AOB	*Actinobacteria*	Total bacteria	Archaea	AOA:AOB
**M (*df = 1*)**	*P = 0.0036*	*P = 0.51*	*P = 0.093*	*P = 0.0671*	*P = 0.17*
**S (*df = 3*)**	*P = 0.53*	*P < 0.001*	*P < 0.0001*	*P < 0.001*	*P = 0.24*
**M×S (*df = 3*)**	*P = 0.78*	*P = 0.69*	*P = 0.65*	*P = 0.63*	*P = 0.47*
