# Supplementary material for: Suppression treatment differentially influences the microbial community and the occurrence of broad host range plasmids in the rhizosphere of the model cover crop Avena sativa L
Source: PLoS One. 2019 Oct 9;14(10):e0223600. doi: 10.1371/journal.pone.0223600 (PMC6785065; doi:10.1371/journal.pone.0223600)
Supplement: S11 Table — The parameter threshold-cycle (Ct) of the amplification curves are indicated for each sample. Standard curve: Ct = 48.33–3.16 log10 (korB copies) (R2 = 0.999; Efficiency = 107.14%). NA indicates no amplification. (PDF) [file pone.0223600.s029.pdf]

Sample identity	Suppression method (M)	Sampling time (S)	Treatment (M×S)	*Ct* parameter
C1.4D	Mowing (C)	4 days (4D)	C.4D	NA
C2.4D	Mowing (C)	4 days (4D)	C.4D	NA
C3.4D	Mowing (C)	4 days (4D)	C.4D	NA
C4.4D	Mowing (C)	4 days (4D)	C.4D	NA
G1.4D	Glyphosate (G)	4 days (4D)	G.4D	33.02
G2.4D	Glyphosate (G)	4 days (4D)	G.4D	NA
G3.4D	Glyphosate (G)	4 days (4D)	G.4D	NA
G4.4D	Glyphosate (G)	4 days (4D)	G.4D	38.05
C1.10D	Mowing (C)	10 days (10D)	C.10D	NA
C2.10D	Mowing (C)	10 days (10D)	C.10D	37.50
C3.10D	Mowing (C)	10 days (10D)	C.10D	NA
C4.10D	Mowing (C)	10 days (10D)	C.10D	38.83
G1.10D	Glyphosate (G)	10 days (10D)	G.10D	NA
G2.10D	Glyphosate (G)	10 days (10D)	G.10D	NA
G3.10D	Glyphosate (G)	10 days (10D)	G.10D	NA
G4.10D	Glyphosate (G)	10 days (10D)	G.10D	NA
C1.17D	Mowing (C)	17 days (17D)	C.17D	NA
C2.17D	Mowing (C)	17 days (17D)	C.17D	NA
C3.17D	Mowing (C)	17 days (17D)	C.17D	34.48
C4.17D	Mowing (C)	17 days (17D)	C.17D	34.27
G1.17D	Glyphosate (G)	17 days (17D)	G.17D	NA
G2.17D	Glyphosate (G)	17 days (17D)	G.17D	NA
G3.17D	Glyphosate(G)	17 days (17D)	G.17D	NA
G4.17D	Glyphosate (G)	17 days (17D)	G.17D	NA
C1.26D	Mowing (C)	26 days (26D)	C.26D	NA
C2.26D	Mowing (C)	26 days (26D)	C.26D	NA
C3.26D	Mowing (C)	26 days (26D)	C.26D	NA
C4.26D	Mowing (C)	26 days (26D)	C.26D	NA
G1.26D	Glyphosate (G)	26 days (26D)	G.26D	NA
G2.26D	Glyphosate (G)	26 days (26D)	G.26D	NA
G3.26D	Glyphosate (G)	26 days (26D)	G.26D	NA
G4.26D	Glyphosate (G)	26 days (26D)	G.26D	NA
Negative control	-	-	-	37.24
